# Finite-time fault-tolerant tracking control for a QUAV with mixed faults and external disturbances based on adaptive global fast terminal sliding mode neural network control method

**DOI:** 10.1038/s41598-025-12110-7

**Published:** 2025-07-26

**Authors:** Xiyu Zhang, Chun Feng, Youjun Zhou, Xiongfeng Deng

**Affiliations:** 1 School of Information Science and Engineering, Xinjiang College of Science & Technology, Korla, 841000 China; 2 School of Mathematics and Computer Science, Guangxi Science and Technology Normal University, Laibin, 546199 China; 3 Key Laboratory of Electric Drive and Control of Anhui Higher Education Institutes, Anhui Polytechnic University, Wuhu, 241000 China

**Keywords:** QUAV, Finite-time control, Mixed faults, Fault-tolerant control, Neural network, Aerospace engineering, Applied mathematics

## Abstract

This paper addresses the finite-time tracking control problem for a class of quadrotor unmanned aerial vehicle (QUAV) subject to unknown mixed faults and external disturbances. The considered mixed faults include both input quantization and actuator faults. First, radial basis function neural networks (RBFNNs) are employed to approximate the unknown nonlinear dynamics of the QUAV system, with adaptive control laws designed for online weights updates. Second, since the neural network approximation errors and external disturbances can be treated as unknown but bounded constants, adaptive control laws are developed to estimate these parameters. Third, to address the design complexity caused by unknown control coefficients arising from mixed faults, a Nussbaum gain function is introduced. Subsequently, based on the designed global fast terminal sliding mode (GFTSM) functions, adaptive GFTSM neural network control strategies are proposed for position and attitude tracking control. Theoretical analysis confirms that these control strategies guarantee the QUAV system’s position and attitude outputs converge to reference trajectories, with tracking errors reaching a very small neighborhood of zero within a finite time. Finally, the effectiveness of proposed control strategies is validated through an actual system.

## Introduction

With the rapid development of control technologies and growing application demands, unmanned aerial vehicles (UAVs) have attracted considerable research significant attention over the past decade. The rise of the low-altitude economy has further elevated the importance and practical value of UAV in modern industries. Among UAVs, QUAVs stand out due to their structural simplicity, agile maneuverability, strong adaptability, and high stability, making them widely applicable in both civilian and military domains^1–3^. To ensure attitude stability and precise trajectory tracking for QUAV system, numerous advanced control strategies have been developed. For instance, literature^4^ proposed an adaptive robust control approach to handle aerodynamic uncertainties, while literature^5,6^ developed an event-triggered control scheme that effectively resolved the stability issue in post-stall pitching maneuver of aircraft. Literature^7^ proposed an adaptive tracking control scheme that combines backstepping control and sliding mode control (SMC), which significantly improves trajectory tracking accuracy and environmental adaptability. Additionally, literature^8^ presented an adaptive prescribed-time control method, ensuring attitude tracking across the entire time domain. Reinforcement learning-based approaches have further enhanced the fault-tolerance capabilities of QUAV system^9,10^. Other notable control schemes, including those in literature^11–13^, have also contributed to this field. Another critical challenge in QUAV control is mitigating external disturbances, such as wind gusts and turbulent airflow. To address these issues, various advanced techniques have been widely adopted, including neural network control methods^8,14,15^, disturbance observers^16,17^, and active disturbance rejection control techniques^12,18,19^ Notably, neural network-based methods proposed in literature^15,20–24^ have proven particularly effective, offering reduced computational complexity and simplified controller design while maintaining high performance. Their efficiency and adaptability make them a promising solution for QUAV control system.

During actual flight operations, the controllers of QUAV system may experience various faults due to architecture limitations, unpredictable flight environments and communication interruptions. If these faults are not dealt with promptly, they may lead to degradation in flight control performance, which will affect the tracking accuracy and may even cause flight accidents. To address these challenges, researchers have developed several advanced fault-tolerant control strategies. For the fault saturation issue, literature^25^ proposed an innovative admittance control-based fault-tolerant method. Regarding input saturation problems, literature^26^ designed an output-feedback fault-tolerant adaptive fuzzy scheme designed, literature^27^ constructed a command filter-based backstepping control strategy constructed, both of which effectively guaranteed the stability of attitude tracking control for the QUAV system. Furthermore, literature^28,29^ investigated the fault-tolerant control problems of QUAV system with actuator faults, where fixed-time tracking control and prescribed-time tracking control were achieved through the application of the designed control strategies, respectively. For comprehensive fault scenarios including sensor, software and hardware faults, literature^30^ developed a data fusion-based controller and literature^31^ proposed an observer-based SMC method, respectively. As can be seen from the above analysis, the proposed advanced control strategies have effectively enhanced the operational safety and reliability of QUAV system under various fault conditions. It should be emphasized that in many of the mentioned works, only one type of fault is considered. However, multiple faults may occur during the operation of QUAV.

On the other hand, it is important to note that controller faults may cause unknown control coefficients, known as the control direction problem. When the control direction of a system is unknown, the design of control laws becomes more challenging, and making the conventional control strategies developed under the assumption of known control directions no longer applicable. Although the fault-tolerant control problems of QUAV system with various faults have been investigated in literature^24–31^, many of these works presuppose known control coefficients, thereby restricting the broader applicability of the proposed methods. Furthermore, communication interruptions can result in discontinuous input signals for QUAV system, a challenge that can be effectively characterized by input quantization. Notably, most existing studies appear to have overlooked this critical aspect. Therefore, it is both necessary and valuable to explore the tracking control problem of QUAV system under conditions of input quantization and actuator faults.

As one of the most effective methods for addressing uncertain dynamics and external disturbances, SMC method has been widely used in QUAV controller design^7,17,20,24,31^. Numerous improved SMC methods have also been developed, as documented in literature^13,19,25,32^. In traditional SMC method, a linear sliding surface is typically employed to ensure that the tracking error gradually converges to zero after the system reaches the sliding mode. However, this approach cannot guarantee the finite-time convergence. To solve this issue, the terminal SMC method is proposed and by researchers. The application of the designed terminal sliding mode controllers has successfully solved the problems of fault-tolerant control^23^, finite-time attitude tracking control^22,33^, and finite-time formation control^34^ of QUAV system. Although the terminal SMC method can ensure the convergence within a finite time, the presence of switching term may cause discontinuous control signals and further lead to the chattering phenomena. To address this issue, literature^35,36^ designed GFTSM control schemes for QUAV system, which can simultaneously guarantee the finite-time convergence for both position and attitude tracking.

Inspired by the aforementioned works, the finite-time fault-tolerant tracking control problem for a QUAV subject to unknown mixed faults and external disturbances is addressed in this paper. By integrating RBFNN, Nussbaum gain function technique and GFTSM control method, adaptive GFTSM neural network control strategies are designed to ensure finite-time convergence in both position and attitude tracking. The main contributions are outlined as follows.

(i) This paper considers a QUAV system affected by unknown mixed faults including actuator faults and input quantization and external disturbances. In contrast to prior studies in literature^24–28^, the proposed model offers a more generalized representation of system faults and disturbances.
(ii) This paper introduces the RNFNN to approximate unknown nonlinear dynamics of the QUAV system, and designs adaptive control laws to achieve neural network weights update and unknown parameters estimation. Additionally, the Nussbaum gain function technique is employed to compensate for unknown control coefficients induced by mixed faults, significantly reducing the complexity of control strategy design.
(iii) By integrating the GFTSM control method with neural network control technique, adaptive GFTSM neural network control strategies are ultimately proposed in this paper. These strategies exhibit exceptional tracking performance, enabling both position and attitude outputs to accurately track desired reference trajectories.
(iv) Despite the presence of unknown mixed faults and external disturbances, the developed control strategies not only ensure that the tracking errors of the QUAV system converge to a small neighborhood of zero within a finite time, but also ensure that all signals of the closed-loop system maintain bounded.

The remainder of this paper is organized as follows. “System statement and preliminaries” section presents the dynamical model of QUAV, along with preliminaries, including the RBFNN and some useful lemmas. “Main results” section details the design process of the position and attitude tracking control strategies, along with stability analysis. In “Simulation analysis” section, a simulation case is given to show the effectiveness of the proposed control method. Finally, the conclusions of this paper are summarized in “Conclusion” section.

### System statement and preliminaries

#### QUAV dynamic model

**Fig. 1:**
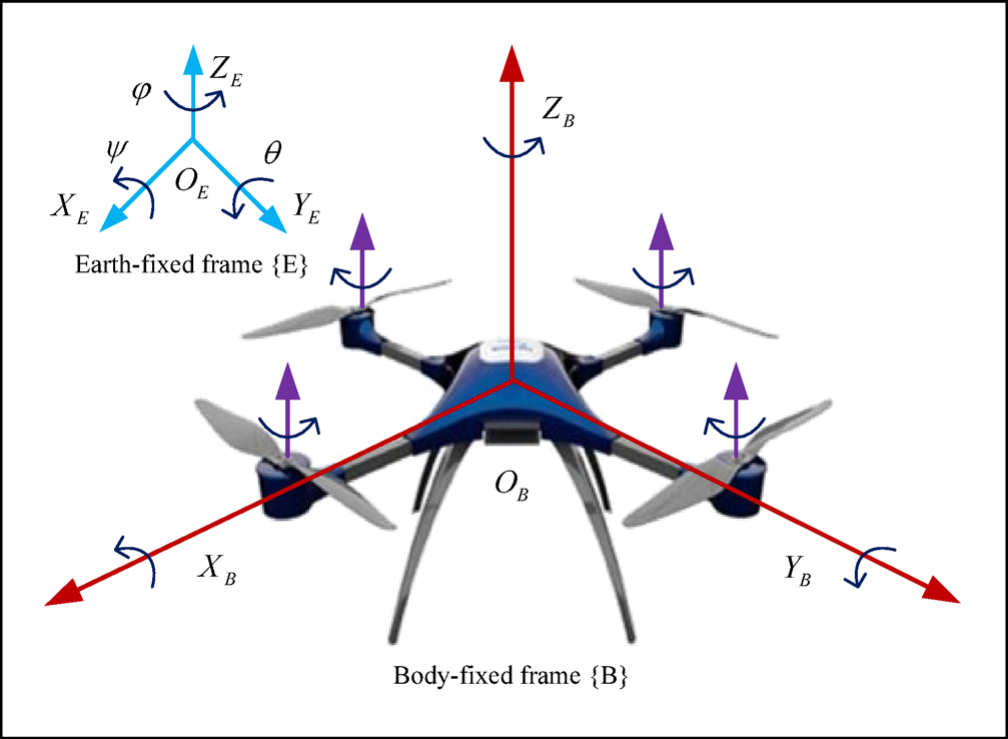
Reference frames of a QUAV.

The dynamic model of QUAV is established with respect to the body-fixed frame $\{ B\}$ and the earth-fixed frame $\{ E\}$, as illustrated in Fig. 1. Prior to deriving the mathematical model of the QUAV, the following necessary assumptions are introduced.

##### Assumption 1

The origin $O_{B}$ of the rigid body coordinate system is aligned with the QUAV’s center of gravity.

##### Assumption 2

The coordinate axes of the rigid body coordinate system $B\{ B_{X} ,B_{Y} ,B_{Z} \}$ coincide with those of the earth coordinate system $E\{ E_{X} ,E_{Y} ,E_{Z} \}$.

Let $x$, $y$ and $z$ stand for the positions of the QUAV on the earth coordinate system, $\theta$, $\psi$ and $\phi$ stand for the pitch angle, yaw angle and roll angle. Meanwhile, the state vector is selected as

$$X = \left[ {x,\dot{x},y,\dot{y},z,\dot{z},\theta ,\dot{\theta },\psi ,\dot{\psi },\phi ,\dot{\phi }} \right]^{T} = \left[ {x_{1} ,x_{2} ,x_{3} ,x_{4} ,x_{5} ,x_{6} ,x_{7} ,x_{8} ,x_{9} ,x_{10} ,x_{11} ,x_{12} } \right]^{T}$$

Building upon the results in literature^5,7,23^ and considering the impact of input quantization and actuator faults, the dynamic model of the QUAV is described as

1 $$\begin{array}{l} {{\dot x}_1} = {x_2} \\  {{\dot x}_2} = \frac{{{\cal Q}({u_1})}}{M}\left( {\sin {x_7}\cos {x_9}\cos {x_{11}} + \sin {x_9}\sin {x_{11}}} \right) - \frac{{{{\cal K}_x}}}{M}{x_2} + {\Delta _x}(t) \\  {{\dot x}_3} = {x_4} \\  {{\dot x}_4} = \frac{{{\cal Q}({u_1})}}{M}\left( {\sin {x_7}\cos {x_9}\sin {x_{11}} - \sin {x_9}\cos {x_{11}}} \right) - \frac{{{{\cal K}_y}}}{M}{x_4} + {\Delta _y}(t) \\  {{\dot x}_5} = {x_6} \\  {{\dot x}_6} = \frac{{{\cal Q}({u_1})}}{M}\left( {\cos {x_9}\cos {x_{11}}} \right) - g - \frac{{{{\cal K}_z}}}{M}{x_6} + {\Delta _z}(t) \\  {{\dot x}_7} = {x_8} \\  {{\dot x}_8} = \frac{1}{{{\cal I}{_y}}}{\cal A}({u_2}) + \frac{{{\cal I}{_z} - {\cal I}{_x}}}{{{\cal I}{_y}}}{x_{10}}{x_{12}} - \frac{{{\cal I}{_r}}}{{{\cal I}{_y}}}{\varpi _r}{x_{12}} - \frac{{{{\cal K}_\theta }}}{{{\cal I}{_x}}}{x_8} + {\Delta _\theta }(t) \\  {{\dot x}_9} = {x_{10}} \\  {{\dot x}_{10}} = \frac{1}{{{\cal I}{_z}}}{\cal A}({u_3}) + \frac{{{\cal I}{_x} - {\cal I}{_y}}}{{{\cal I}{_z}}}{x_8}{x_{12}} - \frac{{{{\cal K}_\psi }}}{{{\cal I}{_x}}}{x_{10}} + {\Delta _\psi }(t) \\  {{\dot x}_{11}} = {x_{12}} \\  {{\dot x}_{12}} = \frac{1}{{{\cal I}{_x}}}{\cal A}({u_4}) + \frac{{{\cal I}{_y} - {\cal I}{_z}}}{{{\cal I}{_x}}}{x_8}{x_{10}} - \frac{{{\cal I}{_r}}}{{{\cal I}{_x}}}{\varpi _r}{x_8} - \frac{{{{\cal K}_\phi }}}{{{\cal I}{_x}}}{x_{12}} + {\Delta _\phi }(t) \\  \end{array}$$

where $M$ is the mass of the QUAV, $g$ is the acceleration of gravity, ${\mathcal{I}}_{r}$ is the moment of inertia of each motor about the coordinate axis, ${\mathcal{I}}_{x}$, ${\mathcal{I}}_{y}$ and ${\mathcal{I}}_{z}$ are the moments of inertia of the three coordinate axes, $\varpi_{r} = w_{1} + w_{2} + w_{3} + w_{4}$ is an air gyro coefficient with $w_{i}$($i = 1,2,3,4$) being the angular velocities of the four rotating propellers, ${\mathcal{K}}_{x}$, ${\mathcal{K}}_{y}$ and ${\mathcal{K}}_{z}$ represent the resistance in three directions, ${\mathcal{K}}_{\theta }$, ${\mathcal{K}}_{\psi }$ and ${\mathcal{K}}_{\phi }$ represent the resistance in three angles, $\Delta_{x} (t)$, $\Delta_{y} (t)$, $\Delta_{z} (t)$, $\Delta_{\theta } (t)$, $\Delta_{\psi } (t)$ and $\Delta_{\phi } (t)$ represent unknown but bounded external disturbances; ${\mathcal{Q}}(u_{1} )$ is the quantized output representing the total lift force, where $u_{1}$ is the input of the quantizer; ${\mathcal{A}}(u_{2} )$, ${\mathcal{A}}(u_{3} )$ and ${\mathcal{A}}(u_{4} )$ are the outputs of actuator faults representing the controllers for pitch motion, yaw motion and roll motion, where $u_{2}$, $u_{3}$ and $u_{4}$ denote the inputs of these actuator faults.

For the QUAV model Eq. (1), the following hysteresis quantizer is used to define ${\mathcal{Q}}(u_{1} )$, that is,

2 $${\mathcal{Q}}(u_{1} ) = \left\{ {\begin{array}{*{20}l} {u_{1m} {\text{sign}}\left( {u_{1} (t)} \right)} \hfill & {\begin{array}{*{20}l} {\frac{{u_{1m} }}{1 + \iota } < \left| {u_{1} (t)} \right| \le u_{1m} ,\;\dot{u}_{1} (t) < 0,\;{\text{or}}} \hfill \\ {u_{1m} < \left| {u_{1} (t)} \right| \le \frac{{u_{1m} }}{1 - \iota },\;\dot{u}_{1} (t) > 0} \hfill \\ \end{array} } \hfill \\ {u_{1m} (1 + \iota ){\text{sign}}\left( {u_{1} (t)} \right)} \hfill & {\begin{array}{*{20}l} {u_{1m} < \left| {u_{1} (t)} \right| \le \frac{{u_{1m} }}{1 - \iota },\;\dot{u}_{1} (t) < 0,\;{\text{or}}} \hfill \\ {\frac{{u_{1m} }}{1 - \iota } < \left| {u_{1} (t)} \right| \le \frac{{u_{1m} (1 + \iota )}}{1 - \iota },\;\dot{u}_{1} (t) > 0} \hfill \\ \end{array} } \hfill \\ 0 \hfill & {\begin{array}{*{20}l} {0 \le \left| {u_{1} (t)} \right| < \frac{{u_{0} }}{1 + \iota },\;\dot{u}_{1} (t) < 0,\;{\text{or}}} \hfill \\ {\frac{{u_{0} }}{1 + \iota } \le \left| {u_{1} (t)} \right| \le u_{0} ,\;\dot{u}_{1} (t) > 0} \hfill \\ \end{array} } \hfill \\ {{\mathcal{Q}}\left( {u_{1} (t^{ - } )} \right)} \hfill & {{\text{otherwise}}} \hfill \\ \end{array} } \right.$$

and the following actuator fault is used to describe ${\mathcal{A}}(u_{i} )$, that is,

3 $${\mathcal{A}}(u_{i} ) = \overset{\lower0.5em\hbox{$\smash{\scriptscriptstyle\frown}$}}{h}_{i} u_{i} (t) + o_{i} (t),\;i = 2,3,4$$

In Eq. (2), $${u_{1m}} = {\mathord{\buildrel{\lower3pt\hbox{$\scriptscriptstyle\frown$}} \over l} ^{(1 - m)}}{u_0}$$, $$m = 1,2, \cdots$$, $$\overset{\lower0.5em\hbox{$\smash{\scriptscriptstyle\frown}$}}{l} \in (0,1)$$ and $$\iota  = (1 - \mathord{\buildrel{\lower3pt\hbox{$\scriptscriptstyle\frown$}} \over l} )/(1 + \mathord{\buildrel{\lower3pt\hbox{$\scriptscriptstyle\frown$}} \over l} )$$, where $$\overset{\lower0.5em\hbox{$\smash{\scriptscriptstyle\frown}$}}{l}$$ is called as the quantization density and $$u_{0} > 0$$ is design parameter. In Eq. (3), $$\overset{\lower0.5em\hbox{$\smash{\scriptscriptstyle\frown}$}}{h}_{i} \in (0,1]$$ represents the unknown lose effectiveness rate, $$o_{i} (t)$$ is a bounded time-varying bias signal and satisfies $$\left| {o_{i} (t)} \right| \le o_{i,M}$$ with $$o_{i,M} > 0$$. Additionally, the hysteresis quantizer $${\mathcal{Q}}(u_{1} )$$ can be rewritten as^37^

4 $${\mathcal{Q}}(u_{1} ) = k(t)u_{1} (t) + \delta (t)$$

where $$k(t) \in [1 - \iota ,1 + \iota ]$$ and $$\left| {\delta (t)} \right| \le u_{0}$$.

As the QUAV is an underactuated system, it cannot simultaneously track all six degrees of freedom. One control scenario considered in this paper is to track positions $x$, $y$ and $z$ as along with the roll angle $\phi$, while simultaneously ensuring the stability of pitch angle $\theta$ and yaw angle $\psi$. Hence, the control objective of this paper is to design adaptive GFTSM neural network fault-tolerant control strategies $u_{i} (t)$($i = 1,2,3,4$) for the QUAV system described by Eq. (1) such that all signals of the closed-loop system are bounded. Meanwhile, the position outputs $x$, $y$ and $z$, and the roll angle output $\phi$ can track the reference trajectories $x_{r}$, $y_{r}$, $z_{r}$ and $\phi_{r}$, while ensuring the tracking errors can converge to zero within a finite time.

##### Assumption 3

The inputs $u_{i} (t)$($i = 1,2,3,4$) satisfy $u_{i} (t) \in \left( {0, + \infty } \right)$, and the pitch angle $\theta$, yaw angle $\psi$ and roll angle $\phi$ of QUAV belong to $[ - {\pi \mathord{\left/ {\vphantom {\pi 2}} \right. \kern-0pt} 2},{\pi \mathord{\left/ {\vphantom {\pi 2}} \right. \kern-0pt} 2}]$.

##### Assumption 4

There exists an unknown positive constant $\Delta_{ * ,M}$ such that $\left| {\Delta_{ * } (t)} \right| \le \Delta_{ * ,M}$, where $*$ represents $x$, $y$, $z$, $\theta$, $\psi$ or $\phi$.

#### RBFNN

According to the results shown in literature^14,22^, any unknown nonlinear function ${\mathcal{Y}}(Z)$ can be approximated by using RBFNN ${\rm M}^{T} \varphi (Z)$, that is

5 $${\mathcal{Y}}(Z) = {\rm M}^{T} \varphi (Z)$$

where $$Z \in \Omega_{Z} \subset R^{n}$$ is the input vector, $${\rm M} = [m_{1} , \cdots ,m_{l} ]^{T} \in R^{l}$$ represents the weight vector, $$\varphi (Z) = \left[ {\varphi_{1} (Z), \cdots ,\varphi_{l} (Z)} \right]^{T} \in R^{l}$$ denotes the basis function vector and $$\varphi_{i} (Z)$$($$i = 1, \cdots ,l$$) are Gaussian functions as $${\varphi _i}(Z) = \exp \!\!\left({\!\!- {{(Z - {{\mathord{\buildrel{\lower3pt\hbox{$\scriptscriptstyle\frown$}} \over \lambda } }_i})}^T}(Z - {{\mathord{\buildrel{\lower3pt\hbox{$\scriptscriptstyle\frown$}} \over \lambda } }_i})/\user2{2}b_i^2} \right)$$ with $$\overset{\lower0.5em\hbox{$\smash{\scriptscriptstyle\frown}$}}{\lambda }_{i} = [\overset{\lower0.5em\hbox{$\smash{\scriptscriptstyle\frown}$}}{\lambda }_{i1} \user2{,} \cdots \user2{,}\overset{\lower0.5em\hbox{$\smash{\scriptscriptstyle\frown}$}}{\lambda }_{in} ]^{T}$$ and $$b_{i}$$ being the center vector and the width, respectively.

Then, the unknown nonlinear function ${\mathcal{F}}(Z)$ over the compact set $\Omega_{Z} \subset R^{n}$ can be approximated as

6 $${\mathcal{F}}(Z) = ({\rm M}^{ * } )^{T} \varphi (Z) + \varepsilon (Z)$$

where $\varepsilon (Z)$ is the approximation error and satisfies $\left| {\varepsilon (Z)} \right| \le \varepsilon_{M}$ with $\varepsilon_{M} > 0$, and the ideal weight vector ${\rm M}^{ * }$ is defined as

7 $${\rm M}^{ * } : = \arg \mathop {\min }\limits_{{{\rm M} \in R^{l} }} \left\{ {\mathop {\sup }\limits_{{Z \in \Omega_{Z} }} \left| {{\mathcal{F}}(Z) - {\rm M}^{T} \varphi (Z)} \right|} \right\}$$

### Useful definition and lemmas

#### Definition 1

^37^. A smooth function ${\mathcal{N}}(\chi )$ which satisfies the following properties

8 $$\left\{ \begin{gathered} \mathop {\lim }\limits_{r \to \infty } \sup \frac{1}{r}\int_{0}^{r} {{\mathcal{N}}(\chi )d\chi } = + \infty \hfill \\ \mathop {\lim }\limits_{r \to \infty } \inf \frac{1}{r}\int_{0}^{r} {{\mathcal{N}}(\chi )d\chi } = - \infty \hfill \\ \end{gathered} \right.$$

is called the Nussbaum-type function. In this paper, the Nussbaum-type function is selected as ${\mathcal{N}}(\chi ) = \exp (\chi^{2} )\cos ({{\pi \chi } \mathord{\left/ {\vphantom {{\pi \chi } 2}} \right. \kern-0pt} 2})$.

#### **Lemma 1**

^38^. *Let* $$\chi_{i} (t)$$ *be smooth function on* $$[0,t_{f} )$$*, and* $$V(t)$$ *be a positive definite function. If the following inequality holds*

9 $$V(t) \le \alpha_{2} + \exp ( - \alpha_{1} t)\sum\limits_{i = 1}^{n} {\int_{0}^{t} {\exp (\alpha_{1} \tau )\left( {{\mathcal{G}}(\tau ){\mathcal{N}}_{i} (\chi_{i} ) + 1} \right)\dot{\chi }_{i} d\tau } }$$

*where* $$\alpha_{1} > 0$$ *and* $$\alpha_{2} > 0$$*,* $${\mathcal{G}}(t)$$ *is non-zero but bounded parameter, and* $${\mathcal{N}}_{i} (\chi_{i} )$$ *represents Nussbaum-type function, then* $$V(t)$$ *and* $$\chi_{i} (t)$$ *are bounded on* $$[0,t_{f} )$$.

#### **Lemma 2**

^39^. *For the GFTSM surface* $s$*, if there exists*

10 $$s = \dot{x} + \gamma x + \beta x^{{{h \mathord{\left/ {\vphantom {h k}} \right. \kern-0pt} k}}} = 0$$

*where* $x \in R$ *is state variable,* $\gamma > 0$ *and* $\beta > 0$ *are design constants,* $0 < h$ *and* $0 < k$ *are positive odd integers and* $0 < h < k$*. Thus, for suitable* $h$ *and* $k$*, and any initial state satisfying* $x(0) \ne 0$*, the system (*10*) will converge to* $x = 0$ *within a finite time* $T_{s}$*, where* $T_{s}$ *satisfies*

11 $$T_{s} = \frac{k}{\gamma (k - h)}\left( {\ln \left( {\gamma x(0)^{{{{(k - h)} \mathord{\left/ {\vphantom {{(k - h)} k}} \right. \kern-0pt} k}}} + \beta } \right) - \ln \beta } \right)$$

#### **Lemma 3**

^40^. *For* ${\mathcal{S}}_{1} \in R$ *and* ${\mathcal{S}}_{2} \in R$*, and any positive constants* $a_{1}$*,* $a_{2}$ *and* $a_{3}$*, the following inequality holds*

12 $$\left| {{\mathcal{S}}_{1} } \right|^{{a_{1} }} \left| {{\mathcal{S}}_{2} } \right|^{{a_{2} }} \le \frac{{a_{1} }}{{a_{1} + a_{2} }}a_{3} \left| {{\mathcal{S}}_{1} } \right|^{{a_{1} + a_{2} }} + \frac{{a_{2} }}{{a_{1} + a_{2} }}a_{3}^{{ - \frac{{a_{1} }}{{a_{2} }}}} \left| {{\mathcal{S}}_{2} } \right|^{{a_{1} + a_{2} }}$$

#### **Lemma 4**

^40^. *For any constants* ${\mathcal{Q}}$ *and* $\varpi > 0$*, the following inequality holds*

13 $$0 \le \left| {\mathcal{Q}} \right| - \frac{{{\mathcal{Q}}^{2} }}{{\sqrt {{\mathcal{Q}}^{2} + \rho^{2} } }} \le \rho$$

## Main results

For the QUAV system with input quantization, actuator faults and external disturbances, this section employs the RBFNN approximation technique, the GFTSM control method and the Nussbaum gain function technique to design both position and attitude tracking control strategies, while also providing the stability analysis process.

### Position tracking control strategy design

*Step 1* Consider the state equation of $X - {\text{axis}}$ position as

14 $$\begin{gathered} \dot{x}_{1} = x_{2} \hfill \\ \dot{x}_{2} = \frac{{{\mathcal{Q}}(u_{1} )}}{M}u_{x} - \frac{{{\mathcal{K}}_{x} }}{M}x_{2} + \Delta_{x} (t) \hfill \\ \end{gathered}$$

where $$u_{x} = \sin x_{7} \cos x_{9} \cos x_{11} + \sin x_{9} \sin x_{11}$$.

Defining the tracking error $e_{x} = x_{1} - x_{r}$ and noting Eq. (4), then the derivative of $e_{x}$ and $\dot{e}_{x}$ are

15 $$\begin{gathered} \dot{e}_{x} = x_{2} - \dot{x}_{r} \hfill \\ \ddot{e}_{x} = g(t)U_{1x} + F_{1} (Z_{1} ) + \Delta_{x} (t) - \ddot{x}_{r} \hfill \\ \end{gathered}$$

where $$g(t) = {{k(t)} \mathord{\left/ {\vphantom {{k(t)} M}} \right. \kern-0pt} M}$$, $$U_{1x} = u_{1} (t)u_{x}$$ and $$F_{1} (Z_{1} ) = {{\delta (t)u_{x} } \mathord{\left/ {\vphantom {{\delta (t)u_{x} } M}} \right. \kern-0pt} M} + {{{\mathcal{K}}_{x} x_{2} } \mathord{\left/ {\vphantom {{{\mathcal{K}}_{x} x_{2} } M}} \right. \kern-0pt} M}$$.

For the nonlinear dynamic $F_{1} (Z_{1} )$ in Eq. (15), an RBFNN is introduced to approximate it, then we have

16 $$F_{1} (Z_{1} ) = ({\rm M}_{1}^{ * } )^{T} \varphi_{1} (Z_{1} ) + \varepsilon_{1} (Z_{1} )$$

where $Z_{1} = \left[ {x_{2} ,x_{7} ,x_{9} ,x_{11} } \right]^{T}$, $\left| {\varepsilon_{1} (Z_{1} )} \right| \le \varepsilon_{1M}$ is approximation error, and $\varepsilon_{1M} > 0$ is unknown constant.

Design the GFTSM function $s_{1}$ as

17 $$s_{1} = \dot{e}_{x} + \gamma_{1} e_{x} + \beta_{1} (e_{x} )^{{{{h_{0} } \mathord{\left/ {\vphantom {{h_{0} } {k_{0} }}} \right. \kern-0pt} {k_{0} }}}}$$

where $\gamma_{1} > 0$ and $\beta_{1} > 0$ are design parameters, $h_{0} > 0$ and $k_{0} > 0$ are odd integers and $0 < {{h_{0} } \mathord{\left/ {\vphantom {{h_{0} } {k_{0} }}} \right. \kern-0pt} {k_{0} }} < 1$.

Considering Eqs. (15) and (16) and let $\mu = {{h_{0} } \mathord{\left/ {\vphantom {{h_{0} } {k_{0} }}} \right. \kern-0pt} {k_{0} }}$, then the derivative of $s_{1}$ is

18 $$\begin{aligned} \dot{s}_{1} & = \ddot{e}_{x} + \gamma_{1} \dot{e}_{x} + \beta_{1} \mu (e_{x} )^{\mu - 1} \dot{e}_{x} \\ & = g(t)U_{1x} + ({\rm M}_{1}^{ * } )^{T} \varphi_{1} (Z_{1} ) + \left( {\varepsilon_{1} (Z_{1} ) + \Delta_{x} (t)} \right) + \gamma_{1} \dot{e}_{x} + \beta_{1} \mu (e_{x} )^{\mu - 1} \dot{e}_{x} - \ddot{x}_{r} \\ \end{aligned}$$

Design the Lyapunov function $V_{1}$ as

19 $$V_{1} = \frac{1}{2}s_{1}^{2} + \frac{1}{{2\sigma_{1} }}\tilde{\Pi }_{1}^{2} + \frac{1}{{2\varsigma_{1} }}\tilde{\rm T}_{1}^{2}$$

where $\sigma_{1} > 0$ and $\varsigma_{1} > 0$ are design parameters, $\tilde{\Pi }_{1} = \Pi_{1} - \hat{\Pi }_{1}$ and $\tilde{\rm T}_{1} = {\rm T}_{1} - \hat{\rm T}_{1}$ represent estimation errors, $\hat{\Pi }_{1}$ and $\hat{\rm T}_{1}$ are the estimations of $\Pi_{1}$ and ${\rm T}_{1}$. Here, $\Pi_{1}$ and ${\rm T}_{1}$ will be given later.

Considering Eq. (18), the derivative of $V_{1}$ is

20 $$\begin{gathered} \dot{V}_{1} = s_{1} \dot{s}_{1} - \frac{1}{{\sigma_{1} }}\tilde{\Pi }_{1} \dot{\hat{\Pi }}_{1} - \frac{1}{{\varsigma_{1} }}\tilde{\rm T}_{1} \dot{\hat{\rm T}}_{1} \hfill \\ \quad = g(t)s_{1} U_{1x} + s_{1} ({\rm M}_{1}^{ * } )^{T} \varphi_{1} (Z_{1} ) + s_{1} \left( {\varepsilon_{1} (Z_{1} ) + \Delta_{x} (t)} \right) + s_{1} \left( {\gamma_{1} \dot{e}_{x} + \beta_{1} \mu (e_{x} )^{\mu - 1} \dot{e}_{x} - \ddot{x}_{r} } \right) \hfill \\ \quad \quad - \frac{1}{{\sigma_{1} }}\tilde{\Pi }_{1} \dot{\hat{\Pi }}_{1} - \frac{1}{{\varsigma_{1} }}\tilde{\rm T}_{1} \dot{\hat{\rm T}}_{1} \hfill \\ \end{gathered}$$

Applying Lemmas 3 and 4, we get

21 $$\left| {s_{1} ({\rm M}_{1}^{ * } )^{T} \varphi_{1} (Z_{1} )} \right| \le d_{1} s_{1}^{2} \Pi_{1} \Upsilon_{1} + \frac{1}{{4d_{1} }}$$

22 $$\left| {s_{1} \left( {\varepsilon_{1} (Z_{1} ) + \Delta_{x} (t)} \right)} \right| \le \left( {\varepsilon_{1M} + \Delta_{x,M} } \right)\left| {s_{1} } \right| \le \frac{{{\rm T}_{1} s_{1}^{2} }}{{\sqrt {s_{1}^{2} + \rho^{2} } }} + {\rm T}_{1} \rho$$

where $d_{1} > 0$ is design parameter, $\Pi_{1} = ({\rm M}_{1}^{ * } )^{T} ({\rm M}_{1}^{ * } )$, $\Upsilon_{1} = \left( {\varphi_{1} (Z_{1} )} \right)^{T} \varphi_{1} (Z_{1} )$ and ${\rm T}_{1} = \varepsilon_{1M} + \Delta_{x,M}$.

Since the gain $g(t) \ne 0$ and is unknown, a Nussbaum function ${\mathcal{N}}_{1} (\chi_{1} )$ is introduced to design the control law $U_{1x}$. Hence, adaptive control laws $\hat{\Pi }_{1}$, $\hat{\rm T}_{1}$ and $\chi_{1}$, and the control law $U_{1x}$ are designed as

23 $$\dot{\hat{\Pi }}_{1} = \sigma_{1} d_{1} s_{1}^{2} \Upsilon_{1} - \upsilon_{1} \hat{\Pi }_{1}$$

24 $$\dot{\hat{\rm T}}_{1} = \frac{{\varsigma_{1} s_{1}^{2} }}{{\sqrt {s_{1}^{2} + \rho^{2} } }} - \vartheta_{1} \hat{\rm T}_{1}$$

25 $$\dot{\chi }_{1} = p_{1} s_{1}^{2} + q_{1} s_{1}^{{\frac{{h_{1} + k_{1} }}{{k_{1} }}}} + d_{1} s_{1}^{2} \hat{\Pi }_{1} \Upsilon_{1} + \frac{{\hat{\rm T}_{1} s_{1}^{2} }}{{\sqrt {s_{1}^{2} + \rho^{2} } }} + s_{1} \left( {\gamma_{1} \dot{e}_{x} + \beta_{1} \mu (e_{x} )^{\mu - 1} \dot{e}_{x} - \ddot{x}_{r} } \right)$$

26 $$U_{1x} = {\mathcal{N}}_{1} (\chi_{1} )\left[ {p_{1} s_{1} + q_{1} s_{1}^{{\frac{{h_{1} }}{{k_{1} }}}} + d_{1} s_{1} \hat{\Pi }_{1} \Upsilon_{1} + \frac{{\hat{\rm T}_{1} s_{1} }}{{\sqrt {s_{1}^{2} + \rho^{2} } }} + \left( {\gamma_{1} \dot{e}_{x} + \beta_{1} \mu (e_{x} )^{\mu - 1} \dot{e}_{x} - \ddot{x}_{r} } \right)} \right]$$

where $\upsilon_{1} > 0$ and $\vartheta_{1} > 0$ are design parameters, $h_{1} > 0$ and $k_{1} > 0$ are odd integers and ${{0 < h_{1} } \mathord{\left/ {\vphantom {{0 < h_{1} } {k_{1} }}} \right. \kern-0pt} {k_{1} }} < 1$.

Substituting Eqs. (21)–(26) into Eq. (20), one has

27 $$\dot{V}_{1} \le \left( {g(t){\mathcal{N}}_{1} (\chi_{1} ) + 1} \right)\dot{\chi }_{1} - p_{1} s_{1}^{2} - q_{1} s_{1}^{{\frac{{h_{1} + k_{1} }}{{k_{1} }}}} + \frac{{\upsilon_{1} }}{{\sigma_{1} }}\tilde{\Pi }_{1} \hat{\Pi }_{1} + \frac{{\vartheta_{1} }}{{\varsigma_{1} }}\tilde{\rm T}_{1} \hat{\rm T}_{1} + \frac{1}{{4d_{1} }} + {\rm T}_{1} \rho$$

*Step 2* Consider the state equation of $Y - {\text{axis}}$ position as

28 $$\begin{gathered} \dot{x}_{3} = x_{4} \hfill \\ \dot{x}_{4} = \frac{{{\mathcal{Q}}(u_{1} )}}{M}u_{y} - \frac{{{\mathcal{K}}_{y} }}{M}x_{4} + \Delta_{y} (t) \hfill \\ \end{gathered}$$

where $$u_{y} = \sin x_{7} \cos x_{9} \sin x_{11} - \sin x_{9} \cos x_{11}$$.

Defining the tracking error $e_{y} = x_{3} - y_{r}$ and noting Eq. (4), then the derivative of $e_{y}$ and $\dot{e}_{y}$ are

29 $$\begin{gathered} \dot{e}_{y} = x_{4} - \dot{y}_{r} \hfill \\ \ddot{e}_{y} = g(t)U_{1y} + F_{2} (Z_{2} ) + \Delta_{y} (t) - \ddot{y}_{r} \hfill \\ \end{gathered}$$

where $$g(t) = {{k(t)} \mathord{\left/ {\vphantom {{k(t)} M}} \right. \kern-0pt} M}$$, $$U_{1y} = u_{1} (t)u_{y}$$ and $$F_{2} (Z_{2} ) = {{\delta (t)u_{y} } \mathord{\left/ {\vphantom {{\delta (t)u_{y} } M}} \right. \kern-0pt} M} + {{{\mathcal{K}}_{y} x_{4} } \mathord{\left/ {\vphantom {{{\mathcal{K}}_{y} x_{4} } M}} \right. \kern-0pt} M}$$.

For the nonlinear dynamic $F_{2} (Z_{2} )$ in Eq. (29), an RBFNN is introduced to approximate it, then we get

30 $$F_{2} (Z_{2} ) = ({\rm M}_{2}^{ * } )^{T} \varphi_{2} (Z_{2} ) + \varepsilon_{2} (Z_{2} )$$

where $Z_{2} = \left[ {x_{4} ,x_{7} ,x_{9} ,x_{11} } \right]^{T}$, $\left| {\varepsilon_{2} (Z_{2} )} \right| \le \varepsilon_{2M}$ is approximation error, and $\varepsilon_{2M} > 0$ is unknown constant.

Design the GFTSM function $s_{2}$ as

31 $$s_{2} = \dot{e}_{y} + \gamma_{2} e_{y} + \beta_{2} (e_{y} )^{\mu }$$

where $\gamma_{2} > 0$ and $\beta_{2} > 0$ are design parameters, $\mu = {{h_{0} } \mathord{\left/ {\vphantom {{h_{0} } {k_{0} }}} \right. \kern-0pt} {k_{0} }}$ is the same as the first step.

Considering Eqs. (29) and (30), then the derivative of $s_{2}$ is

32 $$\begin{gathered} \dot{s}_{2} = \ddot{e}_{y} + \gamma_{2} \dot{e}_{y} + \beta_{2} \mu (e_{y} )^{\mu - 1} \dot{e}_{y} \hfill \\ \quad = g(t)U_{1y} + ({\rm M}_{2}^{ * } )^{T} \varphi_{2} (Z_{2} ) + \left( {\varepsilon_{2} (Z_{2} ) + \Delta_{y} (t)} \right) + \gamma_{2} \dot{e}_{y} + \beta_{2} \mu (e_{y} )^{\mu - 1} \dot{e}_{y} - \ddot{y}_{r} \hfill \\ \end{gathered}$$

Design the Lyapunov function $V_{2}$ as

33 $$V_{2} = \frac{1}{2}s_{2}^{2} + \frac{1}{{2\sigma_{2} }}\tilde{\Pi }_{2}^{2} + \frac{1}{{2\varsigma_{2} }}\tilde{\rm T}_{2}^{2}$$

where $\sigma_{2} > 0$ and $\varsigma_{2} > 0$ are design parameters, $\tilde{\Pi }_{2} = \Pi_{2} - \hat{\Pi }_{2}$ and $\tilde{\rm T}_{2} = {\rm T}_{2} - \hat{\rm T}_{2}$ represent estimation errors, $\hat{\Pi }_{2}$ and $\hat{\rm T}_{2}$ are the estimations of $\Pi_{2}$ and ${\rm T}_{2}$. Here, $\Pi_{2}$ and ${\rm T}_{2}$ will be given later.

Considering Eq. (32), the derivative of $V_{2}$ is

34 $$\begin{gathered} \dot{V}_{2} = s_{2} \dot{s}_{2} - \frac{1}{{\sigma_{1} }}\tilde{\Pi }_{2} \dot{\hat{\Pi }}_{2} - \frac{1}{{\varsigma_{2} }}\tilde{\rm T}_{2} \dot{\hat{\rm T}}_{2} \hfill \\ \quad = g(t)s_{2} U_{1y} + s_{2} ({\rm M}_{2}^{ * } )^{T} \varphi_{2} (Z_{2} ) + s_{2} \left( {\varepsilon_{2} (Z_{2} ) + \Delta_{y} (t)} \right) + s_{2} \left( {\gamma_{2} \dot{e}_{y} + \beta_{2} \mu (e_{y} )^{\mu - 1} \dot{e}_{y} - \ddot{y}_{r} } \right) \hfill \\ \quad \quad - \frac{1}{{\sigma_{2} }}\tilde{\Pi }_{2} \dot{\hat{\Pi }}_{2} - \frac{1}{{\varsigma_{2} }}\tilde{\rm T}_{2} \dot{\hat{\rm T}}_{2} \hfill \\ \end{gathered}$$

Applying Lemmas 3 and 4, we have

35 $$\left| {s_{2} ({\rm M}_{2}^{ * } )^{T} \varphi_{2} (Z_{2} )} \right| \le d_{2} s_{2}^{2} \Pi_{2} \Upsilon_{2} + \frac{1}{{4d_{2} }}$$

36 $$\left| {s_{2} \left( {\varepsilon_{2} (Z_{2} ) + \Delta_{y} (t)} \right)} \right| \le \left( {\varepsilon_{2M} + \Delta_{y,M} } \right)\left| {s_{2} } \right| \le \frac{{{\rm T}_{2} s_{2}^{2} }}{{\sqrt {s_{2}^{2} + \rho^{2} } }} + {\rm T}_{2} \rho$$

where $d_{2} > 0$ is design parameter, $\Pi_{2} = ({\rm M}_{2}^{ * } )^{T} ({\rm M}_{2}^{ * } )$, $\Upsilon_{2} = \left( {\varphi_{2} (Z_{2} )} \right)^{T} \varphi_{2} (Z_{2} )$ and ${\rm T}_{2} = \varepsilon_{2M} + \Delta_{y,M}$.

Since the gain $g(t) \ne 0$ and is unknown, a Nussbaum function ${\mathcal{N}}_{2} (\chi_{2} )$ is introduced to design the control law $U_{1y}$. Hence, adaptive control laws $\hat{\Pi }_{2}$, $\hat{\rm T}_{2}$ and $\chi_{2}$, and the control law $U_{1y}$ are designed as

37 $$\dot{\hat{\Pi }}_{2} = \sigma_{2} d_{2} s_{2}^{2} \Upsilon_{2} - \upsilon_{2} \hat{\Pi }_{2}$$

38 $$\dot{\hat{\rm T}}_{2} = \frac{{\varsigma_{2} s_{2}^{2} }}{{\sqrt {s_{2}^{2} + \rho^{2} } }} - \vartheta_{2} \hat{\rm T}_{2}$$

39 $$\dot{\chi }_{2} = p_{2} s_{2}^{2} + q_{2} s_{2}^{{\frac{{h_{2} + k_{2} }}{{k_{2} }}}} + d_{2} s_{2}^{2} \hat{\Pi }_{2} \Upsilon_{2} + \frac{{\hat{\rm T}_{2} s_{2}^{2} }}{{\sqrt {s_{2}^{2} + \rho^{2} } }} + s_{2} \left( {\gamma_{2} \dot{e}_{y} + \beta_{2} \mu (e_{y} )^{\mu - 1} \dot{e}_{y} - \ddot{y}_{r} } \right)$$

40 $$U_{1y} = {\mathcal{N}}_{2} (\chi_{2} )\left[ {p_{2} s_{2} + q_{2} s_{2}^{{\frac{{h_{2} }}{{k_{2} }}}} + d_{2} s_{2} \hat{\Pi }_{2} \Upsilon_{2} + \frac{{\hat{\rm T}_{2} s_{2} }}{{\sqrt {s_{2}^{2} + \rho^{2} } }} + \left( {\gamma_{2} \dot{e}_{y} + \beta_{2} \mu (e_{y} )^{\mu - 1} \dot{e}_{y} - \ddot{y}_{r} } \right)} \right]$$

where $\upsilon_{2} > 0$ and $\vartheta_{2} > 0$ are design parameters, $h_{2} > 0$ and $k_{2} > 0$ are odd integers and ${{0 < h_{2} } \mathord{\left/ {\vphantom {{0 < h_{2} } {k_{2} }}} \right. \kern-0pt} {k_{2} }} < 1$.

Substituting Eqs. (35)–(40) into Eq. (34), one gets

41 $$\dot{V}_{2} \le \left( {g(t){\mathcal{N}}_{2} (\chi_{2} ) + 1} \right)\dot{\chi }_{2} - p_{2} s_{2}^{2} - q_{2} s_{2}^{{\frac{{h_{2} + k_{2} }}{{k_{2} }}}} + \frac{{\upsilon_{2} }}{{\sigma_{2} }}\tilde{\Pi }_{2} \hat{\Pi }_{2} + \frac{{\vartheta_{2} }}{{\varsigma_{2} }}\tilde{\rm T}_{2} \hat{\rm T}_{2} + \frac{1}{{4d_{2} }} + {\rm T}_{2} \rho$$

*Step 3* Consider the state equation of $Z - {\text{axis}}$ position as

42 $$\begin{gathered} \dot{x}_{5} = x_{6} \hfill \\ \dot{x}_{6} = \frac{{{\mathcal{Q}}(u_{1} )}}{M}u_{z} - g - \frac{{{\mathcal{K}}_{z} }}{M}x_{6} + \Delta_{z} (t) \hfill \\ \end{gathered}$$

where $$u_{z} = \cos x_{9} \cos x_{11}$$.

Defining the tracking error $e_{z} = x_{5} - z_{r}$ and noting Eq. (4), then the derivative of $e_{z}$ and $\dot{e}_{z}$ are

43 $$\begin{gathered} \dot{e}_{z} = x_{6} - \dot{z}_{r} \hfill \\ \ddot{e}_{z} = g(t)U_{1z} + F_{3} (Z_{3} ) + \Delta_{z} (t) - \ddot{z}_{r} \hfill \\ \end{gathered}$$

where $$g(t) = {{k(t)} \mathord{\left/ {\vphantom {{k(t)} M}} \right. \kern-0pt} M}$$, $$U_{1z} = u_{1} (t)u_{z}$$ and $$F_{3} (Z_{3} ) = {{\delta (t)u_{z} } \mathord{\left/ {\vphantom {{\delta (t)u_{z} } M}} \right. \kern-0pt} M} - g + {{{\mathcal{K}}_{z} x_{6} } \mathord{\left/ {\vphantom {{{\mathcal{K}}_{z} x_{6} } M}} \right. \kern-0pt} M}$$.

For the nonlinear dynamic $F_{3} (Z_{3} )$ in Eq. (43), an RBFNN is introduced to approximate it, then we obtain

44 $$F_{3} (Z_{3} ) = ({\rm M}_{3}^{ * } )^{T} \varphi_{3} (Z_{3} ) + \varepsilon_{3} (Z_{3} )$$

where $Z_{3} = \left[ {x_{6} ,x_{9} ,x_{11} } \right]^{T}$, $\left| {\varepsilon_{3} (Z_{3} )} \right| \le \varepsilon_{3M}$ is approximation error, and $\varepsilon_{3M} > 0$ is unknown constant.

Design the GFTSM function $s_{3}$ as

45 $$s_{3} = \dot{e}_{z} + \gamma_{3} e_{z} + \beta_{3} (e_{z} )^{\mu }$$

where $\gamma_{3} > 0$ and $\beta_{3} > 0$ are design parameters, $\mu = {{h_{0} } \mathord{\left/ {\vphantom {{h_{0} } {k_{0} }}} \right. \kern-0pt} {k_{0} }}$ is the same as the first step.

Considering Eqs. (43) and (44), then the derivative of $s_{3}$ is

46 $$\begin{gathered} \dot{s}_{3} = \ddot{e}_{z} + \gamma_{3} \dot{e}_{z} + \beta_{3} \mu (e_{z} )^{\mu - 1} \dot{e}_{z} \hfill \\ \quad = g(t)U_{1z} + ({\rm M}_{3}^{ * } )^{T} \varphi_{3} (Z_{3} ) + \left( {\varepsilon_{3} (Z_{3} ) + \Delta_{z} (t)} \right) + \gamma_{3} \dot{e}_{z} + \beta_{3} \mu (e_{z} )^{\mu - 1} \dot{e}_{z} - \ddot{z}_{r} \hfill \\ \end{gathered}$$

Design the Lyapunov function $V_{3}$ as

47 $$V_{3} = \frac{1}{2}s_{3}^{2} + \frac{1}{{2\sigma_{3} }}\tilde{\Pi }_{3}^{2} + \frac{1}{{2\varsigma_{3} }}\tilde{\rm T}_{3}^{2}$$

where $\sigma_{3} > 0$ and $\varsigma_{3} > 0$ are design parameters, $\tilde{\Pi }_{3} = \Pi_{3} - \hat{\Pi }_{3}$ and $\tilde{\rm T}_{3} = {\rm T}_{3} - \hat{\rm T}_{3}$ represent estimation errors, $\hat{\Pi }_{3}$ and $\hat{\rm T}_{3}$ are the estimations of $\Pi_{3}$ and ${\rm T}_{3}$. Here, $\Pi_{3}$ and ${\rm T}_{3}$ will be given later.

Considering Eq. (46), the derivative of $V_{3}$ is

48 $$\begin{gathered} \dot{V}_{3} = s_{3} \dot{s}_{3} - \frac{1}{{\sigma_{3} }}\tilde{\Pi }_{3} \dot{\hat{\Pi }}_{3} - \frac{1}{{\varsigma_{3} }}\tilde{\rm T}_{3} \dot{\hat{\rm T}}_{3} \hfill \\ \quad = g(t)s_{3} U_{1z} + s_{3} ({\rm M}_{3}^{ * } )^{T} \varphi_{3} (Z_{3} ) + s_{3} \left( {\varepsilon_{3} (Z_{3} ) + \Delta_{z} (t)} \right) + s_{3} \left( {\gamma_{3} \dot{e}_{z} + \beta_{3} \mu (e_{z} )^{\mu - 1} \dot{e}_{z} - \ddot{z}_{r} } \right) \hfill \\ \quad \quad - \frac{1}{{\sigma_{3} }}\tilde{\Pi }_{3} \dot{\hat{\Pi }}_{3} - \frac{1}{{\varsigma_{3} }}\tilde{\rm T}_{3} \dot{\hat{\rm T}}_{3} \hfill \\ \end{gathered}$$

Applying Lemmas 3 and 4, we get

49 $$\left| {s_{3} ({\rm M}_{3}^{ * } )^{T} \varphi_{3} (Z_{3} )} \right| \le d_{3} s_{3}^{2} \Pi_{3} \Upsilon_{3} + \frac{1}{{4d_{3} }}$$

50 $$\left| {s_{3} \left( {\varepsilon_{3} (Z_{3} ) + \Delta_{z} (t)} \right)} \right| \le \left( {\varepsilon_{3M} + \Delta_{z,M} } \right)\left| {s_{3} } \right| \le \frac{{{\rm T}_{3} s_{3}^{2} }}{{\sqrt {s_{3}^{2} + \rho^{2} } }} + {\rm T}_{3} \rho$$

where $d_{3} > 0$ is design parameter, $\Pi_{3} = ({\rm M}_{3}^{ * } )^{T} ({\rm M}_{3}^{ * } )$, $\Upsilon_{3} = \left( {\varphi_{3} (Z_{3} )} \right)^{T} \varphi_{3} (Z_{3} )$ and ${\rm T}_{3} = \varepsilon_{3M} + \Delta_{z,M}$.

Since the gain $g(t) \ne 0$ and is unknown, a Nussbaum function ${\mathcal{N}}_{3} (\chi_{3} )$ is introduced to design the control law $U_{1z}$. Hence, adaptive control laws $\hat{\Pi }_{3}$, $\hat{\rm T}_{3}$ and $\chi_{3}$, and the control law $U_{1z}$ are designed as

51 $$\dot{\hat{\Pi }}_{3} = \sigma_{3} d_{3} s_{3}^{2} \Upsilon_{3} - \upsilon_{3} \hat{\Pi }_{3}$$

52 $$\dot{\hat{\rm T}}_{3} = \frac{{\varsigma_{3} s_{3}^{2} }}{{\sqrt {s_{3}^{2} + \rho^{2} } }} - \vartheta_{3} \hat{\rm T}_{3}$$

53 $$\dot{\chi }_{3} = p_{3} s_{3}^{2} + q_{3} s_{3}^{{\frac{{h_{3} + k_{3} }}{{k_{3} }}}} + d_{3} s_{3}^{2} \hat{\Pi }_{3} \Upsilon_{3} + \frac{{\hat{\rm T}_{3} s_{3}^{2} }}{{\sqrt {s_{3}^{2} + \rho^{2} } }} + s_{3} \left( {\gamma_{3} \dot{e}_{z} + \beta_{3} \mu (e_{z} )^{\mu - 1} \dot{e}_{z} - \ddot{z}_{r} } \right)$$

54 $$U_{1z} = {\mathcal{N}}_{3} (\chi_{3} )\left[ {p_{3} s_{3} + q_{3} s_{3}^{{\frac{{h_{3} }}{{k_{3} }}}} + d_{3} s_{3} \hat{\Pi }_{3} \Upsilon_{3} + \frac{{\hat{\rm T}_{3} s_{3} }}{{\sqrt {s_{3}^{2} + \rho^{2} } }} + \left( {\gamma_{3} \dot{e}_{z} + \beta_{3} \mu (e_{z} )^{\mu - 1} \dot{e}_{z} - \ddot{z}_{r} } \right)} \right]$$

where $\upsilon_{3} > 0$ and $\vartheta_{3} > 0$ are design parameters, $h_{3} > 0$ and $k_{3} > 0$ are odd integers and ${{0 < h_{3} } \mathord{\left/ {\vphantom {{0 < h_{3} } {k_{3} }}} \right. \kern-0pt} {k_{3} }} < 1$.

Substituting Eqs. (49)–(54) into Eq. (58), one has

55 $$\dot{V}_{3} \le \left( {g(t){\mathcal{N}}_{3} (\chi_{3} ) + 1} \right)\dot{\chi }_{3} - p_{3} s_{3}^{2} - q_{3} s_{3}^{{\frac{{h_{3} + k_{3} }}{{k_{3} }}}} + \frac{{\upsilon_{3} }}{{\sigma_{3} }}\tilde{\Pi }_{3} \hat{\Pi }_{3} + \frac{{\vartheta_{3} }}{{\varsigma_{3} }}\tilde{\rm T}_{3} \hat{\rm T}_{3} + \frac{1}{{4d_{3} }} + {\rm T}_{3} \rho$$

Note that $U_{1x} = u_{1} (t)u_{x}$ and $U_{1y} = u_{1} (t)u_{y}$, then the adaptive GFTSM neural network fault-tolerant control strategy $u_{1} (t)$ is derived as

56 $$u_{1} (t) = \left( {U_{1x} \sin x_{11} - U_{1y} \cos x_{11} } \right)\left( {\sin x_{9} } \right)^{ - 1}$$

In view of Assumption 3, it can be got that $\sin x_{9} \ne 0$, so the singularity issue of $u_{1} (t)$ can be avoided.

### Solution of virtual attitude angle

Since the pitch angle $\theta$ and the yaw angle $\psi$ do not have specified reference angles $\theta_{r}$ and $\psi_{r}$, solving for $\theta_{r}$ and $\psi_{r}$ is necessary to achieve tracking of these two angles. Assuming the required pitch and yaw angles for $U_{1x}$ and $U_{1y}$ are $\theta_{r}$ and $\psi_{r}$, respectively, they can be calculated through the following transformation.

Noting the reference trajectory of roll angle is $\phi_{r}$, as well as $U_{1x} = u_{1} (t)u_{x}$ and $U_{1y} = u_{1} (t)u_{y}$, then we obtain

57 $$\left[ {\begin{array}{*{20}c} {U_{1x} } \\ {U_{1y} } \\ \end{array} } \right] = \left[ {\begin{array}{*{20}c} {\sin \theta_{r} \cos \psi_{r} \cos \phi_{r} + \sin \psi_{r} \sin \phi_{r} } \\ {\sin \theta_{r} \cos \psi_{r} \sin \phi_{r} - \sin \psi_{r} \cos \phi_{r} } \\ \end{array} } \right]u_{1} (t) = \left[ {\begin{array}{*{20}c} {\cos \phi_{r} } & {\sin \phi_{r} } \\ {\sin \phi_{r} } & { - \cos \phi_{r} } \\ \end{array} } \right]\left[ {\begin{array}{*{20}c} {\sin \theta_{r} \cos \psi_{r} } \\ {\sin \psi_{r} } \\ \end{array} } \right]u_{1} (t)$$

Considering $u_{z} = \cos \psi_{r} \cos \phi_{r}$ and $U_{1z} = u_{1} (t)u_{z}$, and in view of the fact that

58 $$\left[ {\begin{array}{*{20}c} {\cos \phi_{r} } & {\sin \phi_{r} } \\ {\sin \phi_{r} } & { - \cos \phi_{r} } \\ \end{array} } \right]^{ - 1} = \left[ {\begin{array}{*{20}c} {\cos \phi_{r} } & {\sin \phi_{r} } \\ {\sin \phi_{r} } & { - \cos \phi_{r} } \\ \end{array} } \right]$$

Thus, substituting Eq. (58) into Eq. (57) yields

59 $$\frac{{U_{1z} }}{{\cos \psi_{r} \cos \phi_{r} }}\left[ {\begin{array}{*{20}c} {\sin \theta_{r} \cos \psi_{r} } \\ {\sin \psi_{r} } \\ \end{array} } \right] = \left[ {\begin{array}{*{20}c} {\cos \phi_{r} } & {\sin \phi_{r} } \\ {\sin \phi_{r} } & { - \cos \phi_{r} } \\ \end{array} } \right]\left[ {\begin{array}{*{20}c} {U_{1x} } \\ {U_{1y} } \\ \end{array} } \right]$$

According to the second line of Eq. (59), we obtain

60 $$\tan \psi_{r} = \frac{{\left( {U_{1x} \sin \phi_{r} - U_{1y} \cos \phi_{r} } \right)\cos \phi_{r} }}{{U_{1z} }}$$

Hence, the reference yaw angle $\psi_{r}$ is solved as

61 $$\psi_{r} = \arctan \left( {U_{1z}^{ - 1} \left( {U_{1x} \sin \phi_{r} - U_{1y} \cos \phi_{r} } \right)\cos \phi_{r} } \right)$$

Further, according to the first line of Eq. (59), we have

62 $$\sin \theta_{r} = \frac{{\left( {U_{1x} \cos \phi_{r} + U_{1y} \sin \phi_{r} } \right)\cos \phi_{r} }}{{U_{1z} }}$$

Given that the value on the right side of Eq. (62) may exceed $[ - 1,1]$, the reference pitch angle $\theta_{r}$ is determined using the following scheme, that is

63 $$\theta_{r} = \left\{ {\begin{array}{*{20}c} {\frac{\pi }{2}} & {\Xi \ge 1} \\ {\arcsin \left( {U_{1z}^{ - 1} \left( {U_{1x} \cos \phi_{r} + U_{1y} \sin \phi_{r} } \right)\cos \phi_{r} } \right)} & { - 1 < \Xi < 1} \\ { - \frac{\pi }{2}} & {\Xi \le - 1} \\ \end{array} } \right.$$

where $$\Xi = U_{1z}^{ - 1} \left( {U_{1x} \cos \phi_{r} + U_{1y} \sin \phi_{r} } \right)\cos \phi_{r}$$.

### Attitude tracking control strategy design

To achieve the design of attitude tracking control strategy, the reference angles $\theta_{r}$ and $\psi_{r}$ obtained in the previous subsection, as well as the given roll angle $\phi_{r}$, will be applied.

*Step 4* Consider the state equation of pitch angle as

64 $$\begin{array}{l} {{\dot x}_7} = {x_8} \\  {{\dot x}_8} = \frac{1}{{{\cal I}{_y}}}{\cal A}({u_2}) + \frac{{{\cal I}{_z} - {\cal I}{_x}}}{{{\cal I}{_y}}}{x_{10}}{x_{12}} - \frac{{{\cal I}{_r}}}{{{\cal I}{_y}}}{\varpi _r}{x_{12}} - \frac{{{{\cal K}_\theta }}}{{{\cal I}{_x}}}{x_8} + {\Delta _\theta }(t) \\  \end{array}$$

Defining the tracking error $e_{\theta } = x_{7} - \theta_{r}$ and noting Eq. (3), then the derivative of $e_{\theta }$ and $\dot{e}_{\theta }$ are

65 $$\begin{gathered} \dot{e}_{\theta } = x_{8} - \dot{\theta }_{r} \hfill \\ \ddot{e}_{\theta } = g_{2} (t)u_{2} (t) + F_{4} (Z_{4} ) + \Delta_{\theta } (t) - \ddot{\theta }_{r} \hfill \\ \end{gathered}$$

where $${g_2}(t) = {\mathord{\buildrel{\lower3pt\hbox{$\scriptscriptstyle\frown$}} \over h} _2}/{\cal I}{_y}$$ and $${F_4}({Z_4}) = {{{o_2}(t)} \mathord{\left/ {\vphantom {{{o_2}(t)} {{\cal I}{_y}}}} \right. \kern-\nulldelimiterspace} {{\cal I}{_y}}} + {{({\cal I}{_z} - {\cal I}{_x}){x_{10}}{x_{12}}} \mathord{\left/ {\vphantom {{({\cal I}{_z} - {\cal I}{_x}){x_{10}}{x_{12}}} {{\cal I}{_y}}}} \right. \kern-\nulldelimiterspace} {{\cal I}{_y}}} - {{{\cal I}{_r}{\varpi _r}{x_{12}}} \mathord{\left/ {\vphantom {{{\cal I}{_r}{\varpi _r}{x_{12}}} {{\cal I}{_y}}}} \right. \kern-\nulldelimiterspace} {{\cal I}{_y}}} - {{{{\cal K}_\theta }{x_8}} \mathord{\left/ {\vphantom {{{{\cal K}_\theta }{x_8}} {{\cal I}{_x}}}} \right. \kern-\nulldelimiterspace} {{\cal I}{_x}}}$$.

For the nonlinear dynamic $F_{4} (Z_{4} )$ in Eq. (65), an RBF neural network is introduced to approximate it, then we have

66 $$F_{4} (Z_{4} ) = ({\rm M}_{4}^{ * } )^{T} \varphi_{4} (Z_{4} ) + \varepsilon_{4} (Z_{4} )$$

where $Z_{4} = \left[ {x_{8} ,x_{10} ,x_{12} } \right]^{T}$, $\left| {\varepsilon_{4} (Z_{4} )} \right| \le \varepsilon_{4M}$ is approximation error, and $\varepsilon_{4M} > 0$ is unknown constant.

Design the GFTSM function $s_{4}$ as

67 $$s_{4} = \dot{e}_{\theta } + \gamma_{4} e_{\theta } + \beta_{4} (e_{\theta } )^{\mu }$$

where $\gamma_{4} > 0$ and $\beta_{4} > 0$ are design parameters, $\mu = {{h_{0} } \mathord{\left/ {\vphantom {{h_{0} } {k_{0} }}} \right. \kern-0pt} {k_{0} }}$ is the same as the first step.

Considering Eqs. (65) and (66), then the derivative of $s_{4}$ is

68 $$\begin{gathered} \dot{s}_{4} = \ddot{e}_{\theta } + \gamma_{4} \dot{e}_{\theta } + \beta_{4} \mu (e_{\theta } )^{\mu - 1} \dot{e}_{\theta } \hfill \\ \quad = g_{2} (t)u_{2} (t) + ({\rm M}_{4}^{ * } )^{T} \varphi_{4} (Z_{4} ) + \left( {\varepsilon_{4} (Z_{4} ) + \Delta_{\theta } (t)} \right) + \gamma_{4} \dot{e}_{\theta } + \beta_{4} \mu (e_{\theta } )^{\mu - 1} \dot{e}_{\theta } - \ddot{\theta }_{r} \hfill \\ \end{gathered}$$

Design the Lyapunov function $V_{4}$ as

69 $$V_{4} = \frac{1}{2}s_{4}^{2} + \frac{1}{{2\sigma_{4} }}\tilde{\Pi }_{4}^{2} + \frac{1}{{2\varsigma_{4} }}\tilde{\rm T}_{4}^{2}$$

where $\sigma_{4} > 0$ and $\varsigma_{4} > 0$ are design parameters, $\tilde{\Pi }_{4} = \Pi_{4} - \hat{\Pi }_{4}$ and $\tilde{\rm T}_{4} = {\rm T}_{4} - \hat{\rm T}_{4}$ represent estimation errors, $\hat{\Pi }_{4}$ and $\hat{\rm T}_{4}$ are the estimations of $\Pi_{4}$ and ${\rm T}_{4}$. Here, $\Pi_{4}$ and ${\rm T}_{4}$ will be given later.

Considering Eq. (68), the derivative of $V_{4}$ is

70 $$\begin{gathered} \dot{V}_{4} = s_{4} \dot{s}_{4} - \frac{1}{{\sigma_{4} }}\tilde{\Pi }_{4} \dot{\hat{\Pi }}_{4} - \frac{1}{{\varsigma_{4} }}\tilde{\rm T}_{4} \dot{\hat{\rm T}}_{4} \hfill \\ \quad = g_{2} (t)s_{4} u_{2} (t) + s_{4} ({\rm M}_{4}^{ * } )^{T} \varphi_{4} (Z_{4} ) + s_{4} \left( {\varepsilon_{4} (Z_{4} ) + \Delta_{\theta } (t)} \right) + s_{4} \left( {\gamma_{4} \dot{e}_{\theta } + \beta_{4} \mu (e_{\theta } )^{\mu - 1} \dot{e}_{\theta } - \ddot{\theta }_{r} } \right) \hfill \\ \quad \quad - \frac{1}{{\sigma_{4} }}\tilde{\Pi }_{4} \dot{\hat{\Pi }}_{4} - \frac{1}{{\varsigma_{4} }}\tilde{\rm T}_{4} \dot{\hat{\rm T}}_{4} \hfill \\ \end{gathered}$$

Applying Lemmas 3 and 4, we get

71 $$\left| {s_{4} ({\rm M}_{4}^{ * } )^{T} \varphi_{4} (Z_{4} )} \right| \le d_{4} s_{4}^{2} \Pi_{4} \Upsilon_{4} + \frac{1}{{4d_{4} }}$$

72 $$\left| {s_{4} \left( {\varepsilon_{4} (Z_{4} ) + \Delta_{\theta } (t)} \right)} \right| \le \left( {\varepsilon_{4M} + \Delta_{\theta ,M} } \right)\left| {s_{4} } \right| \le \frac{{{\rm T}_{4} s_{4}^{2} }}{{\sqrt {s_{4}^{2} + \rho^{2} } }} + {\rm T}_{4} \rho$$

where $d_{4} > 0$ is design parameter, $\Pi_{4} = ({\rm M}_{4}^{ * } )^{T} ({\rm M}_{4}^{ * } )$, $\Upsilon_{4} = \left( {\varphi_{4} (Z_{4} )} \right)^{T} \varphi_{4} (Z_{4} )$ and ${\rm T}_{4} = \varepsilon_{4M} + \Delta_{\theta ,M}$.

Since the gain $g_{2} (t) \ne 0$ and is unknown, a Nussbaum function ${\mathcal{N}}_{4} (\chi_{4} )$ is introduced to design the control law $u_{2} (t)$. Hence, adaptive control laws $\hat{\Pi }_{4}$, $\hat{\rm T}_{4}$ and $\chi_{4}$, and the adaptive GFTSM neural network fault-tolerant control strategy $u_{2} (t)$ are designed as

73 $$\dot{\hat{\Pi }}_{4} = \sigma_{4} d_{4} s_{4}^{2} \Upsilon_{4} - \upsilon_{4} \hat{\Pi }_{4}$$

74 $$\dot{\hat{\rm T}}_{4} = \frac{{\varsigma_{4} s_{4}^{2} }}{{\sqrt {s_{4}^{2} + \rho^{2} } }} - \vartheta_{4} \hat{\rm T}_{4}$$

75 $$\dot{\chi }_{4} = p_{4} s_{4}^{2} + q_{4} s_{4}^{{\frac{{h_{4} + k_{4} }}{{k_{4} }}}} + d_{4} s_{4}^{2} \hat{\Pi }_{4} \Upsilon_{4} + \frac{{\hat{\rm T}_{4} s_{4}^{2} }}{{\sqrt {s_{4}^{2} + \rho^{2} } }} + s_{4} \left( {\gamma_{4} \dot{e}_{\theta } + \beta_{4} \mu (e_{\theta } )^{\mu - 1} \dot{e}_{\theta } - \ddot{\theta }_{r} } \right)$$

76 $$u_{2} (t) = {\mathcal{N}}_{4} (\chi_{4} )\left[ {p_{4} s_{4} + q_{4} s_{4}^{{\frac{{h_{4} }}{{k_{4} }}}} + d_{4} s_{4} \hat{\Pi }_{4} \Upsilon_{4} + \frac{{\hat{\rm T}_{4} s_{4} }}{{\sqrt {s_{4}^{2} + \rho^{2} } }} + \left( {\gamma_{4} \dot{e}_{\theta } + \beta_{4} \mu (e_{\theta } )^{\mu - 1} \dot{e}_{\theta } - \ddot{\theta }_{r} } \right)} \right]$$

where $\upsilon_{4} > 0$ and $\vartheta_{4} > 0$ are design parameters, $h_{4} > 0$ and $k_{4} > 0$ are odd integers and $0 < {{h_{4} } \mathord{\left/ {\vphantom {{h_{4} } {k_{4} }}} \right. \kern-0pt} {k_{4} }} < 1$.

Substituting Eqs. (71)–(76) into Eq. (70), one has

77 $$\dot{V}_{4} \le \left( {g_{2} (t){\mathcal{N}}_{4} (\chi_{4} ) + 1} \right)\dot{\chi }_{4} - p_{4} s_{4}^{2} - q_{4} s_{4}^{{\frac{{h_{4} + k_{4} }}{{k_{4} }}}} + \frac{{\upsilon_{4} }}{{\sigma_{4} }}\tilde{\Pi }_{4} \hat{\Pi }_{4} + \frac{{\vartheta_{4} }}{{\varsigma_{4} }}\tilde{\rm T}_{4} \hat{\rm T}_{4} + \frac{1}{{4d_{4} }} + {\rm T}_{4} \rho$$

*Step 5* Consider the state equation of yaw angle as

78 $$\begin{array}{l} {{\dot x}_9} = {x_{10}} \\  {{\dot x}_{10}} = \frac{1}{{{\cal I}{_z}}}{\cal A}({u_3}) + \frac{{{\cal I}{_x} - {\cal I}{_y}}}{{{\cal I}{_z}}}{x_8}{x_{12}} - \frac{{{{\cal K}_\psi }}}{{{\cal I}{_x}}}{x_{10}} + {\Delta _\psi }(t) \\  \end{array}$$

Defining the tracking error $e_{\psi } = x_{9} - \psi_{r}$ and noting Eq. (3), then the derivative of $e_{\psi }$ and $\dot{e}_{\psi }$ are

79 $$\begin{gathered} \dot{e}_{\psi } = x_{10} - \dot{\psi }_{r} \hfill \\ \ddot{e}_{\psi } = g_{3} (t)u_{3} (t) + F_{5} (Z_{5} ) + \Delta_{\psi } (t) - \ddot{\psi }_{r} \hfill \\ \end{gathered}$$

where $${g_3}(t) = {\mathord{\buildrel{\lower3pt\hbox{$\scriptscriptstyle\frown$}} \over h} _3}/{\cal I}{_z}$$ and $${F_5}({Z_5}) = {{{o_3}(t)} \mathord{\left/ {\vphantom {{{o_3}(t)} {{\cal I}{_z}}}} \right. \kern-\nulldelimiterspace} {{\cal I}{_z}}} + {{({\cal I}{_x} - {\cal I}{_y}){x_8}{x_{12}}} \mathord{\left/ {\vphantom {{({\cal I}{_x} - {\cal I}{_y}){x_8}{x_{12}}} {{\cal I}{_z}}}} \right. \kern-\nulldelimiterspace} {{\cal I}{_z}}} - {{{{\cal K}_\psi }{x_{10}}} \mathord{\left/ {\vphantom {{{{\cal K}_\psi }{x_{10}}} {{\cal I}{_x}}}} \right. \kern-\nulldelimiterspace} {{\cal I}{_x}}}$$.

For the nonlinear dynamic $F_{5} (Z_{5} )$ in Eq. (79), an RBF neural network is introduced to approximate it, then we obtain

80 $$F_{5} (Z_{5} ) = ({\rm M}_{5}^{ * } )^{T} \varphi_{5} (Z_{5} ) + \varepsilon_{5} (Z_{5} )$$

where $Z_{5} = \left[ {x_{8} ,x_{10} ,x_{12} } \right]^{T}$, $\left| {\varepsilon_{5} (Z_{5} )} \right| \le \varepsilon_{5M}$ is approximation error, and $\varepsilon_{5M} > 0$ is unknown constant.

Design the GFTSM function $s_{5}$ as

81 $$s_{5} = \dot{e}_{\psi } + \gamma_{5} e_{\psi } + \beta_{5} (e_{\psi } )^{\mu }$$

where $\gamma_{4} > 0$ and $\beta_{4} > 0$ are design parameters, $\mu = {{h_{0} } \mathord{\left/ {\vphantom {{h_{0} } {k_{0} }}} \right. \kern-0pt} {k_{0} }}$ is the same as the first step.

Considering Eqs. (79) and (80), then the derivative of $s_{5}$ is

82 $$\begin{gathered} \dot{s}_{5} = \ddot{e}_{\psi } + \gamma_{5} \dot{e}_{\psi } + \beta_{5} \mu (e_{\psi } )^{\mu - 1} \dot{e}_{\psi } \hfill \\ \quad = g_{3} (t)u_{3} (t) + ({\rm M}_{5}^{ * } )^{T} \varphi_{5} (Z_{5} ) + \left( {\varepsilon_{5} (Z_{5} ) + \Delta_{\psi } (t)} \right) + \gamma_{5} \dot{e}_{\psi } + \beta_{5} \mu (e_{\psi } )^{\mu - 1} \dot{e}_{\psi } - \ddot{\psi }_{r} \hfill \\ \end{gathered}$$

Design the Lyapunov function $V_{5}$ as

83 $$V_{5} = \frac{1}{2}s_{5}^{2} + \frac{1}{{2\sigma_{5} }}\tilde{\Pi }_{5}^{2} + \frac{1}{{2\varsigma_{5} }}\tilde{\rm T}_{5}^{2}$$

where $\sigma_{5} > 0$ and $\varsigma_{5} > 0$ are design parameters, $\tilde{\Pi }_{5} = \Pi_{5} - \hat{\Pi }_{5}$ and $\tilde{\rm T}_{5} = {\rm T}_{5} - \hat{\rm T}_{5}$ represent estimation errors, $\hat{\Pi }_{5}$ and $\hat{\rm T}_{5}$ are the estimations of $\Pi_{5}$ and ${\rm T}_{5}$. Here, $\Pi_{5}$ and ${\rm T}_{5}$ will be given later.

Considering Eq. (82), the derivative of $V_{5}$ is

84 $$\begin{gathered} \dot{V}_{5} = s_{5} \dot{s}_{5} - \frac{1}{{\sigma_{5} }}\tilde{\Pi }_{5} \dot{\hat{\Pi }}_{5} - \frac{1}{{\varsigma_{5} }}\tilde{\rm T}_{5} \dot{\hat{\rm T}}_{5} \hfill \\ \quad = g_{3} (t)s_{5} u_{3} (t) + s_{5} ({\rm M}_{5}^{ * } )^{T} \varphi_{5} (Z_{5} ) + s_{5} \left( {\varepsilon_{5} (Z_{5} ) + \Delta_{\psi } (t)} \right) + s_{5} \left( {\gamma_{5} \dot{e}_{\psi } + \beta_{5} \mu (e_{\psi } )^{\mu - 1} \dot{e}_{\psi } - \ddot{\psi }_{r} } \right) \hfill \\ \quad \quad - \frac{1}{{\sigma_{5} }}\tilde{\Pi }_{5} \dot{\hat{\Pi }}_{5} - \frac{1}{{\varsigma_{5} }}\tilde{\rm T}_{5} \dot{\hat{\rm T}}_{5} \hfill \\ \end{gathered}$$

Applying Lemmas 3 and 4, we get

85 $$\left| {s_{5} ({\rm M}_{5}^{ * } )^{T} \varphi_{5} (Z_{5} )} \right| \le d_{5} s_{5}^{2} \Pi_{5} \Upsilon_{5} + \frac{1}{{4d_{5} }}$$

86 $$\left| {s_{5} \left( {\varepsilon_{5} (Z_{5} ) + \Delta_{\psi } (t)} \right)} \right| \le \left( {\varepsilon_{5M} + \Delta_{\psi ,M} } \right)\left| {s_{5} } \right| \le \frac{{{\rm T}_{5} s_{5}^{2} }}{{\sqrt {s_{5}^{2} + \rho^{2} } }} + {\rm T}_{5} \rho$$

where $d_{5} > 0$ is design parameter, $\Pi_{5} = ({\rm M}_{5}^{ * } )^{T} ({\rm M}_{5}^{ * } )$, $\Upsilon_{5} = \left( {\varphi_{5} (Z_{5} )} \right)^{T} \varphi_{5} (Z_{5} )$ and ${\rm T}_{5} = \varepsilon_{5M} + \Delta_{\psi ,M}$.

Since the gain $g_{3} (t) \ne 0$ and is unknown, a Nussbaum function ${\mathcal{N}}_{5} (\chi_{5} )$ is introduced to design the control law $u_{3} (t)$. Hence, adaptive control laws $\hat{\Pi }_{5}$, $\hat{\rm T}_{5}$ and $\chi_{5}$, and the adaptive GFTSM neural network fault-tolerant control strategy $u_{3} (t)$ as

87 $$\dot{\hat{\Pi }}_{5} = \sigma_{5} d_{5} s_{5}^{2} \Upsilon_{5} - \upsilon_{5} \hat{\Pi }_{5}$$

88 $$\dot{\hat{\rm T}}_{5} = \frac{{\varsigma_{5} s_{5}^{2} }}{{\sqrt {s_{5}^{2} + \rho^{2} } }} - \vartheta_{5} \hat{\rm T}_{5}$$

89 $$\dot{\chi }_{5} = p_{5} s_{5}^{2} + q_{5} s_{5}^{{\frac{{h_{5} + k_{5} }}{{k_{5} }}}} + d_{5} s_{5}^{2} \hat{\Pi }_{5} \Upsilon_{5} + \frac{{\hat{\rm T}_{5} s_{5}^{2} }}{{\sqrt {s_{5}^{2} + \rho^{2} } }} + s_{5} \left( {\gamma_{5} \dot{e}_{\psi } + \beta_{5} \mu (e_{\psi } )^{\mu - 1} \dot{e}_{\psi } - \ddot{\psi }_{r} } \right)$$

90 $$u_{3} (t) = {\mathcal{N}}_{5} (\chi_{5} )\left[ {p_{5} s_{5} + q_{5} s_{5}^{{\frac{{h_{5} }}{{k_{5} }}}} + d_{5} s_{5} \hat{\Pi }_{5} \Upsilon_{5} + \frac{{\hat{\rm T}_{5} s_{5} }}{{\sqrt {s_{5}^{2} + \rho^{2} } }} + \left( {\gamma_{5} \dot{e}_{\psi } + \beta_{5} \mu (e_{\psi } )^{\mu - 1} \dot{e}_{\psi } - \ddot{\psi }_{r} } \right)} \right]$$

where $\upsilon_{5} > 0$ and $\vartheta_{5} > 0$ are design parameters, $h_{5} > 0$ and $k_{5} > 0$ are odd integers and ${{0 < h_{5} } \mathord{\left/ {\vphantom {{0 < h_{5} } {k_{5} }}} \right. \kern-0pt} {k_{5} }} < 1$.

Substituting Eqs. (85)–(90) into Eq. (84), one gets

91 $$\dot{V}_{5} \le \left( {g_{3} (t){\mathcal{N}}_{5} (\chi_{5} ) + 1} \right)\dot{\chi }_{5} - p_{5} s_{5}^{2} - q_{5} s_{5}^{{\frac{{h_{5} + k_{5} }}{{k_{5} }}}} + \frac{{\upsilon_{5} }}{{\sigma_{5} }}\tilde{\Pi }_{5} \hat{\Pi }_{5} + \frac{{\vartheta_{5} }}{{\varsigma_{5} }}\tilde{\rm T}_{5} \hat{\rm T}_{5} + \frac{1}{{4d_{5} }} + {\rm T}_{5} \rho$$

*Step 6* Consider the state equation of roll angle as

92 $$\begin{array}{l} {{\dot x}_{11}} = {x_{12}} \\  {{\dot x}_{12}} = \frac{1}{{{\cal I}{_x}}}{\cal A}({u_4}) + \frac{{{\cal I}{_y} - {\cal I}{_z}}}{{{\cal I}{_x}}}{x_8}{x_{10}} - \frac{{{\cal I}{_r}}}{{{\cal I}{_x}}}{\varpi _r}{x_8} - \frac{{{{\cal K}_\phi }}}{{{\cal I}{_x}}}{x_{12}} + {\Delta _\phi }(t) \\  \end{array}$$

Defining the tracking error $e_{\phi } = x_{11} - \phi_{r}$ and noting Eq. (3), then the derivative of $e_{\phi }$ and $\dot{e}_{\phi }$ are

93 $$\begin{gathered} \dot{e}_{\phi } = x_{12} - \dot{\phi }_{r} \hfill \\ \ddot{e}_{\phi } = g_{4} (t)u_{4} (t) + F_{6} (Z_{6} ) + \Delta_{\phi } (t) - \ddot{\phi }_{r} \hfill \\ \end{gathered}$$

where $${g_4}(t) = {\mathord{\buildrel{\lower3pt\hbox{$\scriptscriptstyle\frown$}} \over h} _4}/{\cal I}{_x}$$ and $${F_6}({Z_6}) = {{{o_4}(t)} \mathord{\left/ {\vphantom {{{o_4}(t)} {{\cal I}{_x}}}} \right. \kern-\nulldelimiterspace} {{\cal I}{_x}}} + {{({\cal I}{_y} - {\cal I}{_z}){x_8}{x_{10}}} \mathord{\left/ {\vphantom {{({\cal I}{_y} - {\cal I}{_z}){x_8}{x_{10}}} {{\cal I}{_x}}}} \right. \kern-\nulldelimiterspace} {{\cal I}{_x}}} - {{{\cal I}{_r}{\varpi _r}{x_8}} \mathord{\left/ {\vphantom {{{\cal I}{_r}{\varpi _r}{x_8}} {{\cal I}{_x}}}} \right. \kern-\nulldelimiterspace} {{\cal I}{_x}}} - {{{{\cal K}_\phi }{x_{12}}} \mathord{\left/ {\vphantom {{{{\cal K}_\phi }{x_{12}}} {{\cal I}{_x}}}} \right. \kern-\nulldelimiterspace} {{\cal I}{_x}}}$$.

For the nonlinear dynamic $F_{6} (Z_{6} )$ in Eq. (93), an RBF neural network is introduced to approximate it, then we have

94 $$F_{6} (Z_{6} ) = ({\rm M}_{6}^{ * } )^{T} \varphi_{6} (Z_{6} ) + \varepsilon_{6} (Z_{6} )$$

where $Z_{6} = \left[ {x_{8} ,x_{10} ,x_{12} } \right]^{T}$, $\left| {\varepsilon_{6} (Z_{6} )} \right| \le \varepsilon_{6M}$ is approximation error, and $\varepsilon_{6M} > 0$ is unknown constant.

Design the GFTSM function $s_{6}$ as

95 $$s_{6} = \dot{e}_{\phi } + \gamma_{6} e_{\phi } + \beta_{6} (e_{\phi } )^{\mu }$$

where $\gamma_{6} > 0$ and $\beta_{6} > 0$ are design parameters, $\mu = {{h_{0} } \mathord{\left/ {\vphantom {{h_{0} } {k_{0} }}} \right. \kern-0pt} {k_{0} }}$ is the same as the first step.

Considering Eqs. (93) and (94), then the derivative of $s_{6}$ is

96 $$\begin{gathered} \dot{s}_{6} = \ddot{e}_{\theta } + \gamma_{6} \dot{e}_{\phi } + \beta_{6} \mu (e_{\phi } )^{\mu - 1} \dot{e}_{\phi } \hfill \\ \quad = g_{4} (t)u_{4} (t) + ({\rm M}_{6}^{ * } )^{T} \varphi_{6} (Z_{6} ) + \left( {\varepsilon_{6} (Z_{6} ) + \Delta_{\phi } (t)} \right) + \gamma_{6} \dot{e}_{\phi } + \beta_{6} \mu (e_{\phi } )^{\mu - 1} \dot{e}_{\phi } - \ddot{\phi }_{r} \hfill \\ \end{gathered}$$

Design the Lyapunov function $V_{6}$ as

97 $$V_{6} = \frac{1}{2}s_{6}^{2} + \frac{1}{{2\sigma_{6} }}\tilde{\Pi }_{6}^{2} + \frac{1}{{2\varsigma_{6} }}\tilde{\rm T}_{6}^{2}$$

where $\sigma_{6} > 0$ and $\varsigma_{6} > 0$ are design parameters, $\tilde{\Pi }_{6} = \Pi_{6} - \hat{\Pi }_{6}$ and $\tilde{\rm T}_{6} = {\rm T}_{6} - \hat{\rm T}_{6}$ represent estimation errors, $\hat{\Pi }_{6}$ and $\hat{\rm T}_{6}$ are the estimations of $\Pi_{6}$ and ${\rm T}_{6}$. Here, $\Pi_{6}$ and ${\rm T}_{6}$ will be given later.

Considering Eq. (96), the derivative of $V_{6}$ is

98 $$\begin{gathered} \dot{V}_{6} = s_{6} \dot{s}_{6} - \frac{1}{{\sigma_{6} }}\tilde{\Pi }_{6} \dot{\hat{\Pi }}_{6} - \frac{1}{{\varsigma_{6} }}\tilde{\rm T}_{6} \dot{\hat{\rm T}}_{6} \hfill \\ \quad = g_{4} (t)s_{6} u_{4} (t) + s_{6} ({\rm M}_{6}^{ * } )^{T} \varphi_{6} (Z_{6} ) + s_{6} \left( {\varepsilon_{6} (Z_{6} ) + \Delta_{\phi } (t)} \right) + s_{6} \left( {\gamma_{6} \dot{e}_{\phi } + \beta_{6} \mu (e_{\phi } )^{\mu - 1} \dot{e}_{\phi } - \ddot{\phi }_{r} } \right) \hfill \\ \quad \quad - \frac{1}{{\sigma_{6} }}\tilde{\Pi }_{6} \dot{\hat{\Pi }}_{6} - \frac{1}{{\varsigma_{6} }}\tilde{\rm T}_{6} \dot{\hat{\rm T}}_{6} \hfill \\ \end{gathered}$$

Applying Lemmas 3 and 4, we get

99 $$\left| {s_{6} ({\rm M}_{6}^{ * } )^{T} \varphi_{6} (Z_{6} )} \right| \le d_{6} s_{6}^{2} \Pi_{6} \Upsilon_{6} + \frac{1}{{4d_{6} }}$$

100 $$\left| {s_{6} \left( {\varepsilon_{6} (Z_{6} ) + \Delta_{\phi } (t)} \right)} \right| \le \left( {\varepsilon_{6M} + \Delta_{\phi ,M} } \right)\left| {s_{6} } \right| \le \frac{{{\rm T}_{6} s_{6}^{2} }}{{\sqrt {s_{6}^{2} + \rho^{2} } }} + {\rm T}_{6} \rho$$

where $d_{6} > 0$ is design parameter, $\Pi_{6} = ({\rm M}_{6}^{ * } )^{T} ({\rm M}_{6}^{ * } )$, $\Upsilon_{6} = \left( {\varphi_{6} (Z_{6} )} \right)^{T} \varphi_{6} (Z_{6} )$ and ${\rm T}_{6} = \varepsilon_{6M} + \Delta_{\phi ,M}$.

Since the gain $g_{4} (t) \ne 0$ and is unknown, a Nussbaum function ${\mathcal{N}}_{6} (\chi_{6} )$ is introduced to design the control law $u_{4} (t)$. Hence, adaptive control laws $\hat{\Pi }_{6}$, $\hat{\rm T}_{6}$ and $\chi_{6}$, and the adaptive GFTSM neural network fault-tolerant control strategy $u_{4} (t)$ are designed as

101 $$\dot{\hat{\Pi }}_{6} = \sigma_{6} d_{6} s_{6}^{2} \Upsilon_{6} - \upsilon_{6} \hat{\Pi }_{6}$$

102 $$\dot{\hat{\rm T}}_{6} = \frac{{\varsigma_{6} s_{6}^{2} }}{{\sqrt {s_{6}^{2} + \rho^{2} } }} - \vartheta_{6} \hat{\rm T}_{6}$$

103 $$\dot{\chi }_{6} = p_{6} s_{6}^{2} + q_{6} s_{6}^{{\frac{{h_{6} + k_{6} }}{{k_{6} }}}} + d_{6} s_{6}^{2} \hat{\Pi }_{6} \Upsilon_{6} + \frac{{\hat{\rm T}_{6} s_{6}^{2} }}{{\sqrt {s_{6}^{2} + \rho^{2} } }} + s_{6} \left( {\gamma_{6} \dot{e}_{\phi } + \beta_{6} \mu (e_{\phi } )^{\mu - 1} \dot{e}_{\phi } - \ddot{\phi }_{r} } \right)$$

104 $$u_{4} (t) = {\mathcal{N}}_{6} (\chi_{6} )\left[ {p_{6} s_{6} + q_{6} s_{6}^{{\frac{{h_{6} }}{{k_{6} }}}} + d_{6} s_{6} \hat{\Pi }_{6} \Upsilon_{6} + \frac{{\hat{\rm T}_{6} s_{6} }}{{\sqrt {s_{6}^{2} + \rho^{2} } }} + \left( {\gamma_{6} \dot{e}_{\phi } + \beta_{6} \mu (e_{\phi } )^{\mu - 1} \dot{e}_{\phi } - \ddot{\phi }_{r} } \right)} \right]$$

where $\upsilon_{6} > 0$ and $\vartheta_{6} > 0$ are design parameters, $h_{6} > 0$ and $k_{6} > 0$ are odd integers and $0 < {{h_{6} } \mathord{\left/ {\vphantom {{h_{6} } {k_{6} }}} \right. \kern-0pt} {k_{6} }} < 1$.

Substituting Eqs. (99)–(104) into Eq. (98) yields

105 $$\dot{V}_{6} \le \left( {g_{4} (t){\mathcal{N}}_{6} (\chi_{6} ) + 1} \right)\dot{\chi }_{6} - p_{6} s_{6}^{2} - q_{6} s_{6}^{{\frac{{h_{6} + k_{6} }}{{k_{6} }}}} + \frac{{\upsilon_{6} }}{{\sigma_{6} }}\tilde{\Pi }_{6} \hat{\Pi }_{6} + \frac{{\vartheta_{6} }}{{\varsigma_{6} }}\tilde{\rm T}_{6} \hat{\rm T}_{6} + \frac{1}{{4d_{6} }} + {\rm T}_{6} \rho$$

### Stability analysis

In view of the results of the above discussion, the main work of this paper can be summarized as the following theorem.

#### **Theorem 1**

*Consider the QUAV system Eq. (*1*) with input quantization, actuator faults and external disturbances, under Assumptions* 3*and* 4*, the adaptive control laws Eqs. (*23*)–(*25*), (*37*)–(*39*), (*51*)–(*53*), (*73*)–(*75*), (*87*)–(*89*) and Eqs. (*101*)–(*103*), and the adaptive GFTSM neural network fault-tolerant control strategies Eq. (*56*) with control laws Eqs. (*26*) and (*40*), Eq. (*76*), Eq. (*90*) and Eq. (*104*), it can be ensured that all signals of the closed-loop system remain bounded and the tracking errors* $e_{x}$*,* $e_{y}$*,* $e_{z}$*,* $e_{\theta }$*,* $e_{\psi }$ *and* $e_{\phi }$ *can converge to zero within a finite time.*

#### *Proof*

Design the following Lyapunov function $V$ as.

106 $$V = \sum\limits_{i = 1}^{6} {V_{i} }$$

Note that Eqs. (27), (41), (55), (77), (91) and Eq. (105), then the derivative of $V$ is

107 $$\begin{gathered} \dot{V} \le - \sum\limits_{i = 1}^{6} {p_{i} s_{i}^{2} } - \sum\limits_{i = 1}^{6} {q_{i} s_{i}^{{\frac{{h_{i} + k_{i} }}{{k_{i} }}}} } + \sum\limits_{i = 1}^{6} {\frac{{\upsilon_{i} }}{{\sigma_{i} }}\tilde{\Pi }_{i} \hat{\Pi }_{i} } + \sum\limits_{i = 1}^{6} {\frac{{\vartheta_{i} }}{{\varsigma_{i} }}\tilde{\rm T}_{i} \hat{\rm T}_{i} } + \sum\limits_{i = 1}^{6} {\left( {G_{i} (t){\mathcal{N}}_{i} (\chi_{i} ) + 1} \right)\dot{\chi }_{i} } \hfill \\ \quad \;\; + \sum\limits_{i = 1}^{6} {\left( {\frac{1}{{4d_{i} }} + {\rm T}_{i} \rho } \right)} \hfill \\ \end{gathered}$$

where $$G_{1} (t) = G_{2} (t) = G_{3} (t) = g(t)$$, $$G_{4} (t) = g_{2} (t)$$, $$G_{5} (t) = g_{3} (t)$$ and $$G_{6} (t) = g_{4} (t)$$.

Using Lemma 3, we have

108 $$\frac{{\upsilon_{i} }}{{\sigma_{i} }}\tilde{\Pi }_{i} \hat{\Pi }_{i} \le - \frac{{\upsilon_{i} }}{{2\sigma_{i} }}\tilde{\Pi }_{i}^{2} + \frac{{\upsilon_{i} }}{{2\sigma_{i} }}\Pi_{i}^{2}$$

109 $$\frac{{\vartheta_{i} }}{{\varsigma_{i} }}\tilde{\rm T}_{i} \hat{\rm T}_{i} \le - \frac{{\vartheta_{i} }}{{2\varsigma_{i} }}\tilde{\rm T}_{i}^{2} + \frac{{\vartheta_{i} }}{{2\varsigma_{i} }}{\rm T}_{i}^{2}$$

Substituting Eqs. (108) and (109) into Eq. (107), one gets

110 $$\begin{gathered} \dot{V} \le - \sum\limits_{i = 1}^{6} {p_{i} s_{i}^{2} } - \sum\limits_{i = 1}^{6} {q_{i} s_{i}^{{\frac{{h_{i} + k_{i} }}{{k_{i} }}}} } - \sum\limits_{i = 1}^{6} {\frac{{\upsilon_{i} }}{{2\sigma_{i} }}\tilde{\Pi }_{i}^{2} } - \sum\limits_{i = 1}^{6} {\frac{{\vartheta_{i} }}{{2\varsigma_{i} }}\tilde{\rm T}_{i}^{2} } + \sum\limits_{i = 1}^{6} {\left( {G_{i} (t){\mathcal{N}}_{i} (\chi_{i} ) + 1} \right)\dot{\chi }_{i} } + C_{0} \hfill \\ \quad \le - \alpha_{1} V - \sum\limits_{i = 1}^{6} {q_{i} s_{i}^{{\frac{{h_{i} + k_{i} }}{{k_{i} }}}} } + \sum\limits_{i = 1}^{6} {\left( {G_{i} (t){\mathcal{N}}_{i} (\chi_{i} ) + 1} \right)\dot{\chi }_{i} } + C_{0} \hfill \\ \end{gathered}$$

where $$\alpha_{1} = \min \left\{ {2p_{i} ,\upsilon_{i} ,\vartheta_{i} ,i = 1, \cdots ,6} \right\}$$ and $$C_{0} = \sum\nolimits_{i = 1}^{6} {\left( {{1 \mathord{\left/ {\vphantom {1 {4d_{i} }}} \right. \kern-0pt} {4d_{i} }} + {\rm T}_{i} \rho + {{\upsilon_{i} \Pi_{i}^{2} } \mathord{\left/ {\vphantom {{\upsilon_{i} \Pi_{i}^{2} } {2\sigma_{i} }}} \right. \kern-0pt} {2\sigma_{i} }} + {{\vartheta_{i} {\rm T}_{i}^{2} } \mathord{\left/ {\vphantom {{\vartheta_{i} {\rm T}_{i}^{2} } {2\varsigma_{i} }}} \right. \kern-0pt} {2\varsigma_{i} }}} \right)}$$.

Since $h_{i}$ and $k_{i}$($i = 1, \cdots ,6$) are positive odd integers, it can be concluded that $h_{i} + k_{i}$ are even. Thereby, we have $s_{i}^{{{{(h_{i} + k_{i} )} \mathord{\left/ {\vphantom {{(h_{i} + k_{i} )} {k_{i} }}} \right. \kern-0pt} {k_{i} }}}} \ge 0$. From Eq. (110), one has

111 $$\dot{V} \le - \alpha_{1} V + \sum\limits_{i = 1}^{6} {\left( {G_{i} (t){\mathcal{N}}_{i} (\chi_{i} ) + 1} \right)\dot{\chi }_{i} } + C_{0}$$

Next, we will prove the conclusion of this paper through the following two steps.

*Step 1* All signals of the closed-loop system remain bounded.

Multiplying inequality Eq. (111) by $\exp (\alpha_{1} t)$ on both side, and taking the integration over $[0,t]$, one obtains

112 $$\begin{gathered} V(t) \le \frac{{C_{0} }}{{\alpha_{1} }} + \left( {V(0) - \frac{{C_{0} }}{{\alpha_{1} }}} \right)\exp ( - \alpha_{1} t) + \exp ( - \alpha_{1} t)\sum\limits_{i = 1}^{6} {\int_{0}^{t} {\exp (\alpha_{1} \tau )\left( {G_{i} (\tau ){\mathcal{N}}_{i} (\chi_{i} ) + 1} \right)\dot{\chi }_{i} } d\tau } \hfill \\ \quad \quad \le \alpha_{2} + \exp ( - \alpha_{1} t)\sum\limits_{i = 1}^{6} {\int_{0}^{t} {\exp (\alpha_{1} \tau )\left( {G_{i} (\tau ){\mathcal{N}}_{i} (\chi_{i} ) + 1} \right)\dot{\chi }_{i} } d\tau } \hfill \\ \end{gathered}$$

where $$\alpha_{2} = {{C_{0} } \mathord{\left/ {\vphantom {{C_{0} } {\alpha_{1} }}} \right. \kern-0pt} {\alpha_{1} }} + V(0)$$.

According to Lemma 1, it can be obtained that $V(t)$ and $\chi_{i}$ are bounded. Further, in view of the definition of $V(t)$, it is easy to conclude that all signals of the closed-loop system are bounded. The proof of the step is completed.

*Step 2* The tracking error converges to zero within a finite time.

Define the following compact set

113 $$\Omega = \left\{ {\left. {\left( {s_{1} , \cdots ,s_{6} ,\tilde{\Pi }_{1} , \cdots ,\tilde{\Pi }_{6} ,\tilde{\rm T}_{1} , \cdots ,\tilde{\rm T}_{6} } \right)} \right|\sum\limits_{i = 1}^{6} {\left( {s_{i}^{2} + \frac{1}{{\sigma_{i} }}\tilde{\Pi }_{i}^{2} + \frac{1}{{\varsigma_{i} }}\tilde{\rm T}_{i}^{2} } \right) \le 2\Theta } } \right\}$$

where $\Theta > 0$ is an arbitrary constant. Assuming the initial condition of the system satisfies $V(0) \le \Theta$, that is, there exists $V(0) \subset \Omega$. It is not difficult to see that the boundary of $\Omega$ is ${\rm P} = \left\{ {\left. {\left( {s_{1} , \cdots ,s_{6} ,\tilde{\Pi }_{1} , \cdots ,\tilde{\Pi }_{6} ,\tilde{\rm T}_{1} , \cdots ,\tilde{\rm T}_{6} } \right)} \right|V = \Theta } \right\}$.

Since $V(t)$ and $\chi_{i}$ are bounded, according to Eqs. (25), (26), (39), (40), (53), (54), (75), (76), (89), (90), (103) and Eq. (104), as well as the definition of $G_{i} (t)$, it can be concluded that $\sum\nolimits_{i = 1}^{6} {\left( {G_{i} (t){\mathcal{N}}_{i} (\chi_{i} ) + 1} \right)\dot{\chi }_{i} }$ is bounded. Without loss of generality, let $\left| {\sum\nolimits_{i = 1}^{6} {\left( {G_{i} (t){\mathcal{N}}_{i} (\chi_{i} ) + 1} \right)\dot{\chi }_{i} } + C_{0} } \right| \le B_{0}$, then we have

114 $$\dot{V} \le - \alpha_{1} V + B_{0}$$

Further, taking $\alpha_{1} > {{B_{0} } \mathord{\left/ {\vphantom {{B_{0} } \Theta }} \right. \kern-0pt} \Theta }$, and note that $V = \Theta$ on ${\rm P}$, then Eq. (114) can be rewritten as

115 $$\dot{V} \le - \frac{{B_{0} }}{\Theta }\Theta + B_{0} = 0$$

Considering the definition of $V$, when $\dot{V} \equiv 0$, there exists $\sum\nolimits_{i = 1}^{6} {s_{i}^{2} } \equiv 0$. According to the LaSalle invariant principle, we have $s_{i} \to 0$ as $t \to \infty$. Therefore, considering the definition of $s_{i}$($i = 1, \cdots ,6$) and using Lemma 2, it can be obtained that

116 $$\lim_{t \to \infty } e_{ * } = \lim_{t \to \infty } \left( { * - *_{r} } \right) = 0$$

and the finite time $T_{s}^{ * }$ is estimated as

117 $$T_{s}^{ * } = \frac{{k_{0} }}{{\gamma_{i} (k_{0} - h_{0} )}}\ln \left[ {\frac{{\gamma_{i} \left( {V(0)} \right)^{{\frac{{k_{0} - h_{0} }}{{k_{0} }}}} + \beta_{i} }}{{\beta_{i} }}} \right]$$

where $*$ represents $x$, $y$, $z$, $\theta$, $\psi$ or $\phi$. The proof of this step is completed.

By synthesizing the discussion results from the above two steps, Theorem 1 is proven.

#### *Remark 1*

By observing Eqs. (26), (40), (54), (56), (76), (90), and (104), it is not difficult to find that their variations can be achieved by adjusting parameters $h_{0}$, $k_{0}$, $p_{i}$, $q_{i}$, $h_{i}$, $k_{i}$, $d_{i}$, $\gamma_{i}$ and $\beta_{i}$, where $i = 1, \cdots ,6$. However, changes in parameters $h_{0}$, $k_{0}$, $\gamma_{i}$ and $\beta_{i}$ will affect the convergence time indicated by Eq. (117), thereby influencing the system’s convergence performance. Clearly, if the convergence time is too short, it may lead to significant overshoot in the system’s control signals or even cause instability. On the other hand, excessively slow convergence is unfavorable for practical applications. Therefore, a reasonable trade-off between these factors is essential when selecting parameters.

### Simulation analysis

**Fig. 2:**
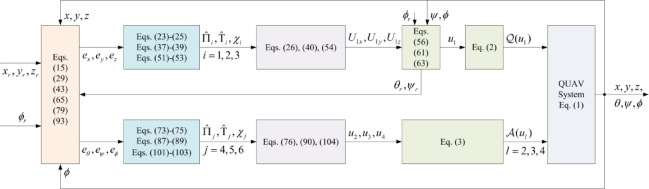
Control block diagram of UAV system.

In this section, a simulation case is provided to illustrate the validity of the proposed control method. The model of the QUAV system is shown in Eq. (1), the initial values are selected as $x(0) = 4.0({\text{m}})$, $y(0) = 2.0({\text{m}})$, $z(0) = 0.0({\text{m}})$, $\dot{x}(0) = \dot{y}(0) = \dot{z}(0) = 0.0({{\text{m}} \mathord{\left/ {\vphantom {{\text{m}} {\text{s}}}} \right. \kern-0pt} {\text{s}}})$, $\theta (0) = \psi (0) = \phi (0) = 0.0({\text{rad}})$, and $\dot{\theta }(0) = \dot{\psi }(0) = \dot{\phi }(0) = 0.0({{{\text{rad}}} \mathord{\left/ {\vphantom {{{\text{rad}}} {\text{s}}}} \right. \kern-0pt} {\text{s}}})$. The reference trajectories are given as $x_{r} = 1.5\left( {1 - \cos (0.5\pi t)} \right)$, $y_{r} = 1.5\sin (0.5\pi t)$ and $z_{r} = 3.0 + 0.5t$. The reference angle $\phi_{r}$ is chosen to be $\phi_{r} = {\pi \mathord{\left/ {\vphantom {\pi 6}} \right. \kern-0pt} 6}$. The reference angles $\theta_{r}$ and $\psi_{r}$ can be obtained by applying Eq. (61) and Eq. (63). The simulation time is $t = 25({\text{s}})$. The control block diagram of QUAV system is displayed in Fig. 2.

The RBFNNs for $$F_{1}$$ and $$F_{2}$$ contain 9 nodes with the centers $$\overset{\lower0.5em\hbox{$\smash{\scriptscriptstyle\frown}$}}{\lambda }_{1}$$ and $$\overset{\lower0.5em\hbox{$\smash{\scriptscriptstyle\frown}$}}{\lambda }_{2}$$ evenly spaced in $$[ - 8,8] \times [ - 8,8] \times [ - 8,8] \times [ - 8,8]$$ and the widths $$b_{1} = b_{2} = 2.0$$, the RBFNNs for $$F_{3}$$ and $$F_{4}$$ contain 9 nodes with the centers $$\overset{\lower0.5em\hbox{$\smash{\scriptscriptstyle\frown}$}}{\lambda }_{3}$$ and $$\overset{\lower0.5em\hbox{$\smash{\scriptscriptstyle\frown}$}}{\lambda }_{4}$$ evenly spaced in $$[ - 8,8] \times [ - 8,8] \times [ - 8,8]$$ and the widths $$b_{3} = b_{4} = 2.0$$, the RBFNN for $$F_{5}$$ contains 9 nodes with the center $$\overset{\lower0.5em\hbox{$\smash{\scriptscriptstyle\frown}$}}{\lambda }_{5}$$ evenly spaced in $$[ - 10,10] \times [ - 10,10] \times [ - 10,10]$$ and the width $$b_{5} = 2.0$$, and the RBFNN for $$F_{6}$$ contains 9 nodes with the center $$\overset{\lower0.5em\hbox{$\smash{\scriptscriptstyle\frown}$}}{\lambda }_{6}$$ evenly spaced in $$[ - 6,6] \times [ - 6,6] \times [ - 6,6]$$ and the width $$b_{6} = 2.0$$. The parameters for the quantizer shown in Eq. (2) are $$u_{0} = 0.05$$, $$\overset{\lower0.5em\hbox{$\smash{\scriptscriptstyle\frown}$}}{l} = 0.2$$, $$\iota = {2 \mathord{\left/ {\vphantom {2 3}} \right. \kern-0pt} 3}$$ and $$m = 100$$, and the parameters for the actuator faults shown in Eq. (3) are given to be $$\overset{\lower0.5em\hbox{$\smash{\scriptscriptstyle\frown}$}}{h}_{2} = \overset{\lower0.5em\hbox{$\smash{\scriptscriptstyle\frown}$}}{h}_{3} = \overset{\lower0.5em\hbox{$\smash{\scriptscriptstyle\frown}$}}{h}_{4} = 0.8$$ and $$o_{2} (t) = o_{3} (t) = o_{4} (t) = 0.25\sin (t)$$ for $$t \ge 4({\text{s)}}$$.

**Fig. 3:**
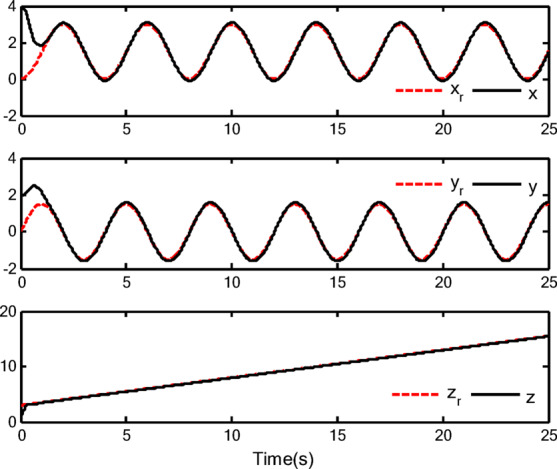
Position tracking results.

**Fig. 4:**
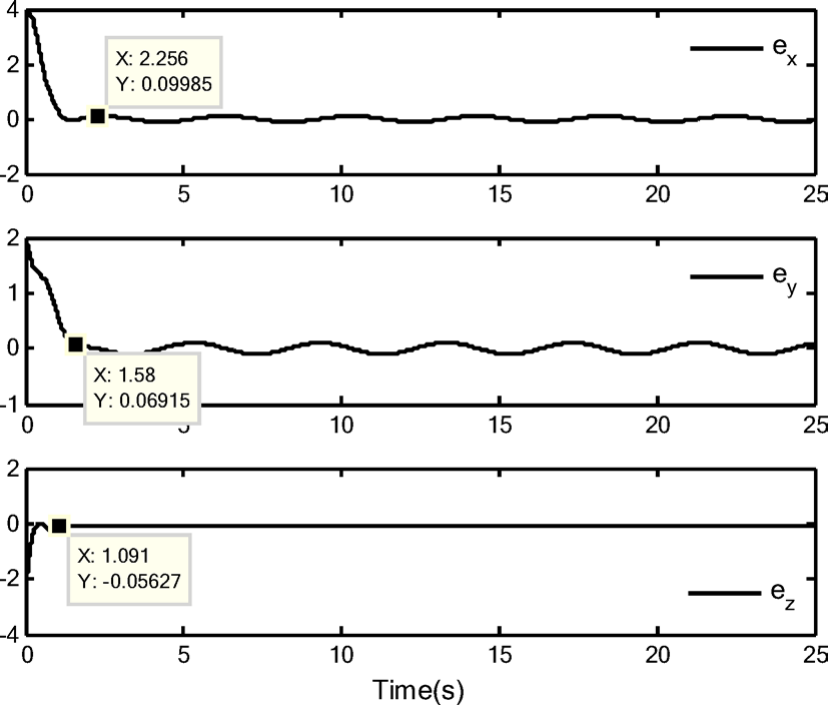
Position tracking errors.

**Fig. 5:**
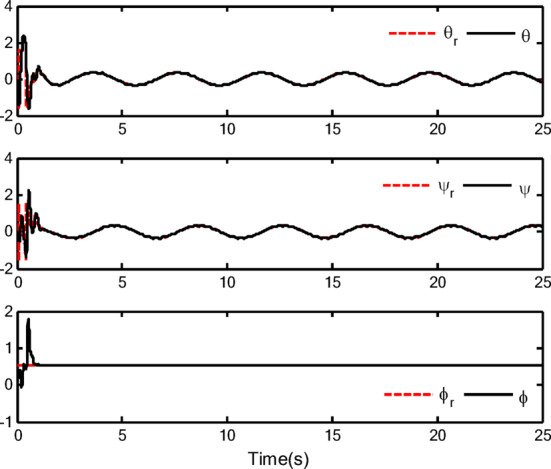
Attitude tracking results.

**Fig. 6:**
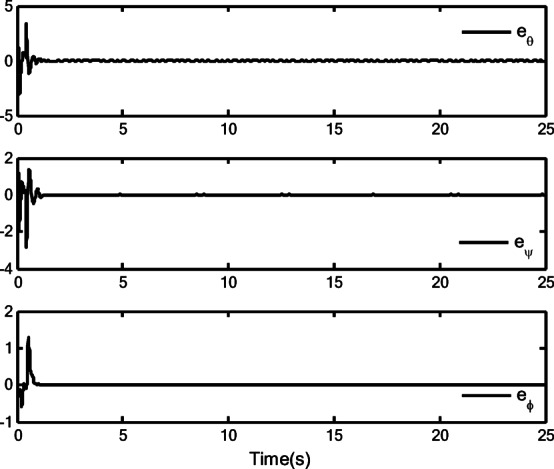
Attitude tracking errors.

**Fig. 7:**
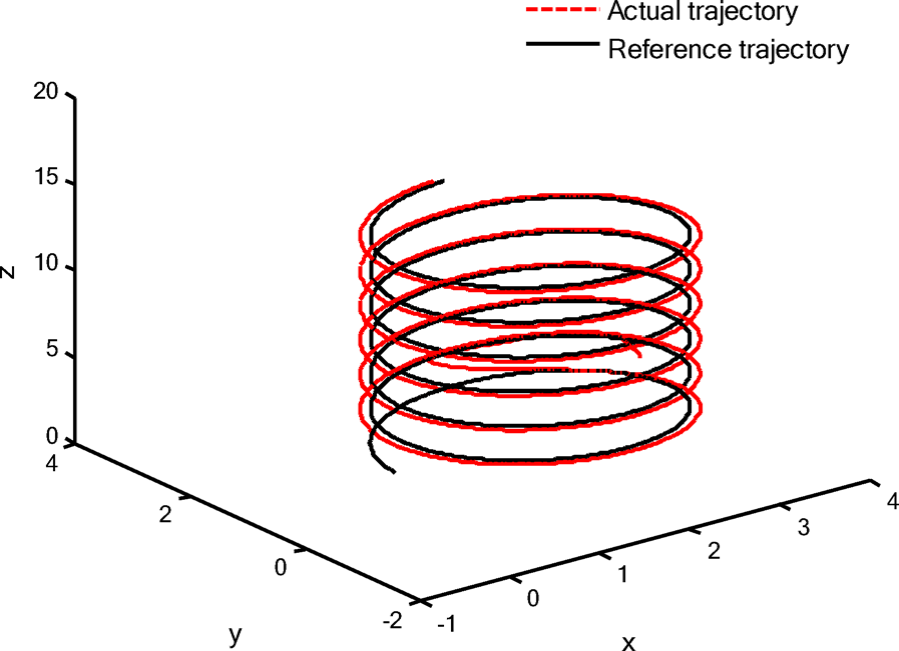
Trajectory tracking result.

**Fig. 8:**
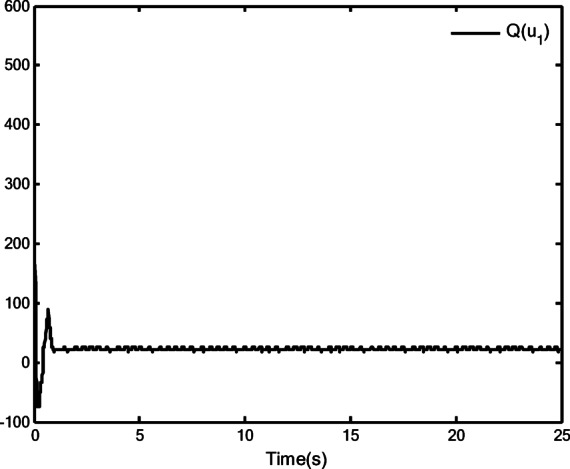
Position control input ${\mathcal{Q}}(u_{1} )$.

**Fig. 9:**
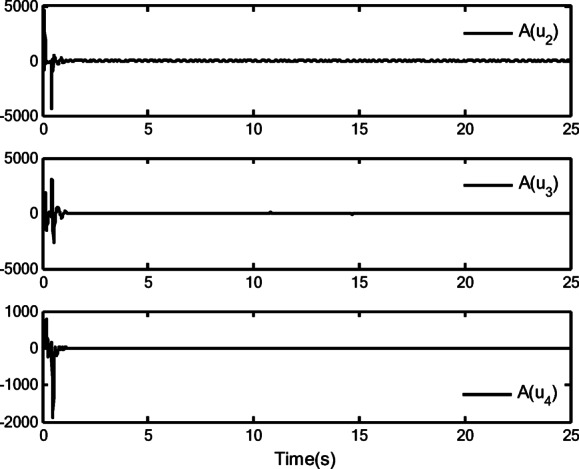
Attitude control inputs ${\mathcal{A}}(u_{2} )$, ${\mathcal{A}}(u_{3} )$ and ${\mathcal{A}}(u_{4} )$.

**Fig. 10:**
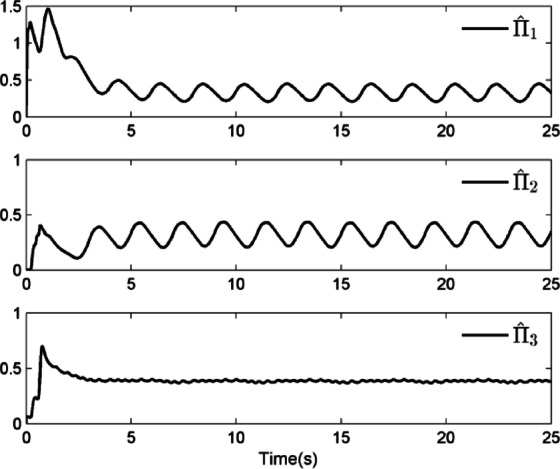
Adaptive control laws $\hat{\Pi }_{1}$, $\hat{\Pi }_{2}$ and $\hat{\Pi }_{3}$ for position tracking.

**Fig. 11:**
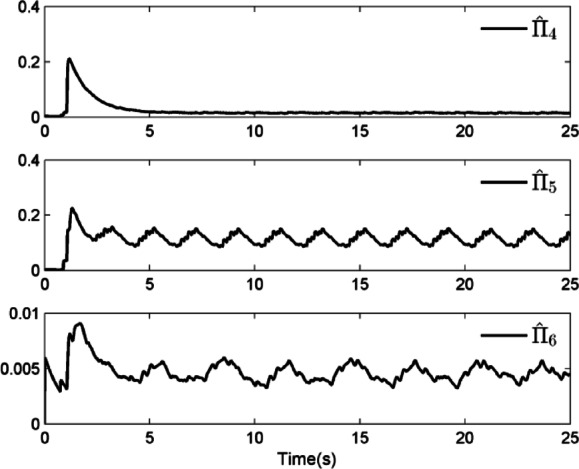
Adaptive control laws $\hat{\Pi }_{4}$, $\hat{\Pi }_{5}$ and $\hat{\Pi }_{6}$ for attitude tracking.

**Fig. 12:**
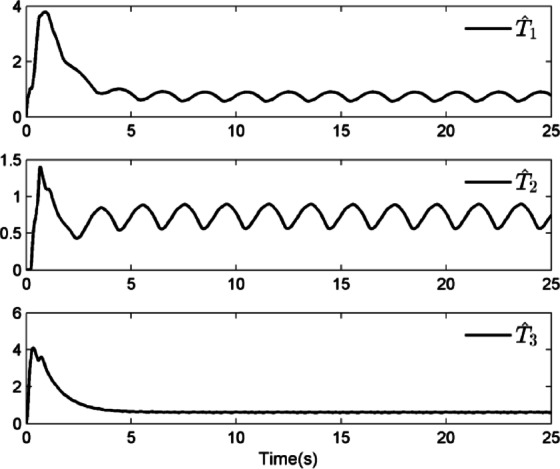
Adaptive control laws $\hat{\rm T}_{1}$, $\hat{\rm T}_{2}$ and $\hat{\rm T}_{3}$ for position tracking.

**Fig. 13:**
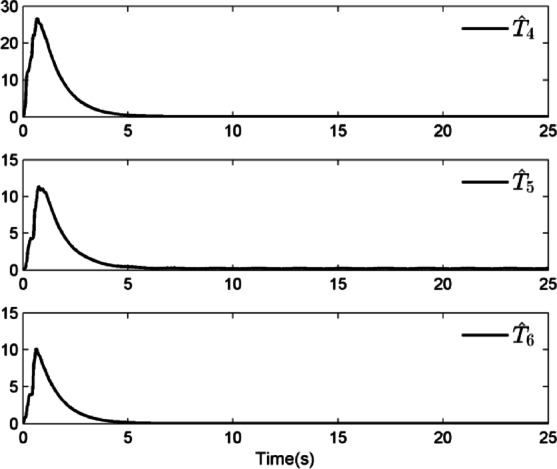
Adaptive control laws $\hat{\rm T}_{4}$, $\hat{\rm T}_{5}$ and $\hat{\rm T}_{6}$ for attitude tracking.

The parameters for QUAV are $M = 2.0({\text{Kg}})$, $g = 9.8({{\text{N}} \mathord{\left/ {\vphantom {{\text{N}} {{\text{Kg}}}}} \right. \kern-0pt} {{\text{Kg}}}})$, $\varpi_{r} = 0.01$, ${\mathcal{I}}_{x} = {\mathcal{I}}_{y} = 1.25$, ${\mathcal{I}}_{z} = 2.5$, ${\mathcal{I}}_{r} = 0.2$,${\mathcal{K}}_{x} = {\mathcal{K}}_{y} = {\mathcal{K}}_{z} = 0.01$, ${\mathcal{K}}_{\theta } = {\mathcal{K}}_{\psi } = {\mathcal{K}}_{\phi } = 0.012$, $\Delta_{x} (t) = \Delta_{y} (t) = \Delta_{z} (t) = 0.01\sin (t)$, and $\Delta_{\theta } (t) = \Delta_{\psi } (t) = \Delta_{\phi } (t) = 0.01\sin (t)$. The parameters for adaptive laws are $h_{0} = 3$, $k_{0} = 7$, $\rho = 1.0$, $\gamma_{i} = 0.2$, $\beta_{i} = 1.5$, $\sigma_{i} = 2.0$, $d_{i} = 1.0$, $\upsilon_{i} = 1.0$, $\varsigma_{i} = 3.5$, $\vartheta_{i} = 1.0$, $p_{i} = 30$, $q_{i} = 11.5$, $h_{i} = 5$, $k_{i} = 9$, and $i = 1, \cdots ,6$. The initial states for adaptive laws are $\hat{\Pi }_{i} (0) = 0.0$, $\hat{\rm T}_{i} (0) = 0.0$, $\chi_{i} (0) = 0.0$, and $i = 1, \cdots ,6$. The simulation results are shown in Figs. 3, 4, 5, 6, 7, 8, 9, 10, 11, 12, 13.

In Figs. 3 and 4, the position tracking performance and the corresponding tracking errors are given, respectively. Furthermore, the attitude tracking performance and the corresponding tracking errors are shown in Figs. 5 and 6, respectively. From these figures, it can be seen that the inputs of QUAV system can effectively track the reference signals, and the tracking errors can converge to a very small range. Despite multiple challenges including input quantization, multiple actuator faults, and external disturbances, the QUAV system maintains excellent tracking performance for both position and attitude when the designed control strategies are applied.

Particularly, it can be seen from Fig. 4 that the three position outputs of the QUAV system can converge to a small neighborhood of zero within a finite time, where the finite times are $T_{s}^{x} = 2.256({\text{s}})$, $T_{s}^{y} = 1.58({\text{s}})$ and $T_{s}^{z} = 1.091({\text{s}})$, respectively. Based on the given initial position states, and design parameters $h_{0}$, $k_{0}$, $\gamma_{i}$ and $\beta_{i}$($i = 1, \cdots ,6$), the theoretical times can be calculated as $T_{s}^{x} = 2.2581({\text{s}})$, $T_{s}^{y} = {1}{\text{.5817}}({\text{s}})$ and $T_{s}^{z} = {1}{\text{.0952}}({\text{s}})$, respectively. Obviously, the actual results are basically consistent with the theoretical results.

Figure 7 displays the curves of the actual trajectory of QUAV system and the reference trajectory. It can be clearly observed that the QUAV system can effectively track the reference trajectory under the proposed control method. However, the tracking error converges only to a very small neighborhood of zero. This limitation arises due to the quantized input signal, actuator faults, and external disturbances, which prevent the actual trajectory from perfectly aligning with the reference trajectory. Although there is a certain error in the tracking process, it just reflects the correctness of the theoretical results.

Figures 8 and 9 depict the control input signals of the QUAV system. Owing to the quantization of input signals and the presence of actuator faults, these signals exhibit non-smooth behavior. Specifically, the signals display large amplitudes during the initial stage of the simulation, but their amplitudes gradually decrease and stabilize within a smaller range as the simulation progresses. These results further underscore the effectiveness of the proposed control method.

The curves of adaptive control laws are given in Figs. 10, 11, 12, 13, respectively. From these figures, it can be observed that these signals exhibited significant overshoot during the initial 5 s of simulation before converging to the steady state. Overall, these signals are ultimately bounded and maintain a very small range.

Based on the simulation results presented above, it is not difficult to see that the control strategies proposed in this paper effectively achieve tracking control of the QUAV system with multiple faults and external disturbances. Meantime, the tracking errors can converge to a very small neighborhood of zero within a finite time. Although all signals of the QUAV system exhibit large amplitudes during the initial stage of the simulation, they are ultimately bounded and maintain a very small range as the simulation progresses. These results clearly demonstrate the efficacy of the proposed control strategies.

## Conclusion

In this paper, the finite-time tracking control problem for a QUAV with unknown mixed faults and external disturbances is discussed. Since the application of the RBFNN and the Nussbaum gain function, the issues include the approximation of unknown nonlinear dynamics and the unknown control coefficients caused by mixed faults are respectively solved. Furthermore, adaptive GFTSM neural network control strategies are proposed to achieve the position and attitude tracking. By applying the proposed control strategies, it can be ensured that the outputs of QUAV system can effectively track the reference trajectories and angles, and the tracking errors can converge to a very small neighborhood of zero within a finite time. The simulation results effectively verify the effectiveness of the proposed method.

This paper primarily discusses the finite-time tracking control problem of QUAV system. In the application of QUAVs, however, the fixed-time control or the predefined-time control is also crucial. When input quantization and actuator faults are present, how to design corresponding controllers to ensure the realization of fixed-time control or predefined-time control is clearly a topic worth exploring. This will be the focus of our future research.

## Data Availability

The data is available from the corresponding author on reasonable request.
